# The response mechanism analysis of *HMX1* knockout strain to levulinic acid in Saccharomyces cerevisiae

**DOI:** 10.3389/fmicb.2024.1416903

**Published:** 2024-06-26

**Authors:** Jiaye Tang, Yulei Chen, Qian Li, Wenli Xin, Ximeng Xiao, Xuemei Chen, Lixi Yang, Borui Mou, Jialian Li, Fujia Lu, Chun Fu, Wencong Long, Hong Liao, Xuebing Han, Peng Feng, Wei Li, Kedi Zhou, Liuyun Yang, Yaojun Yang, Menggen Ma, Hanyu Wang

**Affiliations:** ^1^Bamboo Diseases and Pests Control and Resources Development Key Laboratory of Sichuan Province, College of Life Science, Leshan Normal University, Leshan, Sichuan, China; ^2^College of Resources, Sichuan Agricultural University, Chengdu, Sichuan, China; ^3^College of Bioscience and Biotechnology, Hunan Agricultural University, Changsha, Hunan, China; ^4^Aba Prefecture Ecological Protection and Development Research Institute, Wenchuan, Sichuan, China; ^5^Institute of Nature Conservation Area Planning, Sichuan Forestry and Grassland Survey and Planning Institute, Chengdu, Sichuan, China

**Keywords:** saccharomyces cerevisiae, levulinic acid, response mechanism, transcriptome, metabolome

## Abstract

Levulinic acid, a hydrolysis product of lignocellulose, can be metabolized into important compounds in the field of medicine and pesticides by engineered strains of Saccharomyces cerevisiae. Levulinic acid, as an intermediate product widely found in the conversion process of lignocellulosic biomass, has multiple applications. However, its toxicity to Saccharomyces cerevisiae reduces its conversion efficiency, so screening Saccharomyces cerevisiae genes that can tolerate levulinic acid becomes the key. By creating a whole-genome knockout library and bioinformatics analysis, this study used the phenotypic characteristics of cells as the basis for screening and found the *HMX1* gene that is highly sensitive to levulinic acid in the oxidative stress pathway. After knocking out *HMX1* and treating with levulinic acid, the omics data of the strain revealed that multiple affected pathways, especially the expression of 14 genes related to the cell wall and membrane system, were significantly downregulated. The levels of acetyl-CoA and riboflavin decreased by 1.02-fold and 1.44-fold, respectively, while the content of pantothenic acid increased. These findings indicate that the cell wall-membrane system, as well as the metabolism of acetyl-CoA and riboflavin, are important in improving the resistance of Saccharomyces cerevisiae to levulinic acid. They provide theoretical support for enhancing the tolerance of microorganisms to levulinic acid, which is significant for optimizing the conversion process of lignocellulosic biomass to levulinic acid.

## 1 Introduction

Levulinic acid (LA) is a kind of green platform compound with high additional value derived from the hydrolysis of lignocellulose (Ennaert et al., 2016; Chen and Lee, 2020). LA is a γ-ketonic acid containing five carbon atoms (Pileidis and Titirici, 2016) and has a wide range of applications in fuel additives (Lange et al., 2010; Alonso et al., 2013), liquid fuels, medicine, agriculture, and other fields (Han et al., 2019). Since lignocellulosic biomass can produce LA without the use of expensive hydrolytic enzymes, this greatly reduces the cost of utilizing lignocellulosic biomass. LA can be converted into useful chemicals, such as LA esters, γ-valerolactone (GVL), and alkanes of various molecular weights (Pileidis and Titirici, 2016; Adeleye et al., 2019). Studies have shown that (Habe et al., 2020) certain microorganisms, such as Pseudomonas putida KT2440, Brevibacterium epidermidis LA39-2, *Rhodopseudomonas* sp. No. 7, and Pseudomonas sp. LA18T, can effectively convert LA into polyhydroxyalkanoates (PHA). PHA is a natural macromolecular biomaterial with good biocompatibility, biodegradability, and thermoplastic processing performance similar to plastics. Therefore, it can be used as both a biomedical material and a biodegradable packaging material. In addition, scientists also use engineered brewer's yeast to produce 5-aminolevulinic acid (5-ALA) (Hara et al., 2019) through efficient fermentation in the biosynthetic pathway of heme, which is essential for organisms to synthesize chlorophyll, heme, vitamin B12, and other substances. It is often used in agriculture to increase photosynthesis efficiency and promote color change. In medicine, 5-ALA is a new type of photodynamic drug that is not only used for the treatment of local or generalized skin cancer, but also for the diagnosis of bladder cancer, digestive tract cancer, lung cancer, and other types of cancer.

Despite its ability to produce PHA at a certain level, Pseudomonas putida poses a potential threat to human health due to its pathogenic properties. Therefore, it is of utmost importance to select a healthy and safe fermentation strain. Currently, Saccharomyces cerevisiae stands out as a preferred strain for genetic engineering, owing to its advantages. Furthermore, it is widely acknowledged by the U.S. Food and Drug Administration as a Generally Recognized As Safe (GRAS) microbial strain. Although Saccharomyces cerevisiae lacks the innate ability to directly convert LA into PHA, its straightforward and modifiable genome renders it a prime candidate for genetic engineering. Through this process, it can be transformed into a strain proficient in utilizing LA for PHA production. As an example, researchers have successfully identified and characterized a seven-gene operon in Pseudomonas putida KT2440 that facilitates the catabolism of LA. By introducing this operon into Saccharomyces cerevisiae, a synthetic pathway for PHA production can be established. However, we must acknowledge that technical hurdles persist, primarily stemming from the toxic effects of LA, an acidic compound, on Saccharomyces cerevisiae, which can hinder fermentation efficiency (Li et al., 2017). The toxicity of LA is predominantly exhibited through its ability to induce acidification within the intracellular environment. Cells need to consume ATP to exclude excess protons and maintain intracellular pH stability, which will affect the energy supply of cells (Hyland et al., 2013), and then affect the growth and fermentation process of Saccharomyces cerevisiae. Consequently, a thorough investigation into the tolerance mechanism of Saccharomyces cerevisiae toward LA is imperative for developing yeast strains that are better suited for industrial-scale applications.

Previous studies have shown that the general catabolic repressor Mig1pΔ can enhance the tolerance and fermentation efficiency of Saccharomyces cerevisiae to toxic concentrations of acetic acid, formic acid, and LA (Balderas-Hernández et al., 2018). After adaptive laboratory evolution (ALE), the *S. cerevisiae* F3 strain has gained enhanced tolerance to formic acid and unexpectedly demonstrated higher tolerance to acetic acid. In addition, in yeast cells treated with formic acid, the levels of aromatic amino acids have increased, nucleotide synthesis has slowed down, and energy consumption has decreased. These changes may have enhanced the yeast's tolerance to formic acid. However, the intricate mechanisms that contribute to the LA tolerance in Saccharomyces cerevisiae remain unexplored.

This study aims to leverage the Saccharomyces cerevisiae whole-genome knockout library and SGAtool software to systematically screen for genes that are crucial for LA tolerance. Comprehensive analyses using KEGG and GO pathways, coupled with spot plate experiments, have revealed that the deletion of the *HMX1* gene markedly heightens the sensitivity of Saccharomyces cerevisiae to LA, thereby suggesting that the *HMX1* gene plays a pivotal role in enhancing the yeast's resilience under LA stress. To further elucidate this mechanism, we have employed cutting-edge transcriptomics and metabolomics techniques to unravel the stress response mechanism exhibited by the HMX1 knockout strain in the presence of LA, ultimately confirming the regulatory function of HMX1 in modulating the yeast's tolerance to LA.

## 2 Experimental materials and methods

### 2.1 Fungal strains, culture medium, and culture conditions

The knockout library of non-essential genes of Saccharomyces cerevisiae used in this experiment was donated by Professor Beidong Liu from Gothenburg University. LA, agar, peptone, glucose, yeast extract, geneticin (G418), sodium chloride, etc. were purchased from Chengdu Wanke Co., Ltd. The fluorescent dyes, Mito Tracker Green FM, Yeast Vacuole Membrane Marker MDY-64, and ER-Tracker Red dye, were all purchased from Thermo Scientific, while 2'7'-dichlorofluorescein diacetate was purchased from Sigma. YPD solid medium was prepared by adding 2 g of glucose, 2 g of agar, 1 g of yeast extract, and 2 g of peptone per 100 mL. The YPD liquid medium is prepared by removing 2 g of agar from the above-mentioned basis. YPD+G418 medium was prepared by adding G418 to a final concentration of 100 mg/L to the YPD medium. Before conducting spot plate experiments, the knockout strains were first inoculated on YPD+G418 plates by streaking to obtain single colonies. A single colony was then inoculated into a 100 mL Erlenmeyer flask containing 30 mL of YPD+G418 liquid medium and incubated at 30°C with shaking at 200 r/min for 18–24 h.

### 2.2 Spot plating

The cultured fungal suspensions were uniformly calibrated to a cell concentration (OD_600_) of 1.0 and then diluted in a 10-fold concentration gradient to obtain suspensions of different concentrations. A multi-channel pipette was used to aspirate 5 μL of the diluted fungal suspension and spot it onto YPD+G418 solid medium containing different concentrations of inhibitors. After incubation for 3–4 days, the plates were observed and photographed.

### 2.3 Subcellular structure staining

To investigate the growth rate and survival rate of the gene knockout strain relative to its parent strain BY4741, both strains were overnight cultured in a biological shaker at 30°C and 200 rpm. During this process, the initially cultured strain samples were defined as 0 h. Subsequently, the overnight cultured strains were transferred to liquid YPD+G418 medium containing 100 mM LA (at this point, the OD_600_ value of the samples was 0.8) and further cultured for 3 h under the same conditions. Samples defined as 3 h were also collected during this period. After that, the 0-h and 3-h strain samples were transferred to 1.5 mL EP tubes (at this point, the OD_600_ value of the samples was 1.0). The structural integrity of the cells was observed using a fluorescence confocal microscope equipped with DIC, GFP, Rhod, and DAPI filters. The accumulation of reactive oxygen species, nuclear chromatin disorders, mitochondrial structure, and the morphology of the endoplasmic reticulum and vacuoles in the BY4741 standard strain (non-knockout strain) and the *HMX1* gene knockout strain were evaluated, and the proportions of different morphologies were counted (Madeo et al., 1999).

### 2.4 Screening of LA-tolerant or sensitive knockout strains

After overnight culturing, the strains BY4741 and the *HMX1* gene knockout strain were adjusted to a cell concentration of OD_600_ = 0.1, and 20 mL samples were taken for transcriptome and metabolome analysis, generating the datasets YOK202W_Q_LA and YLR205CC_Q_LA. Subsequently, 100 mM LA was added to the medium, and the cells were further cultured for 3 h. Another 20 mL samples were taken to obtain the datasets YOK202W_H_LA and YLR205C_H_LA. Transcriptome analysis was performed in triplicate, and metabolome analysis was also repeated three times. In addition, the remaining culture was further incubated, and cell density was measured every 6 h to plot a growth curve, assess the impact of gene knockout on strain tolerance, and ensure sampling accuracy. Among them, the datasets YOK202W_Q_LA and YOK202W_H_LA generated from the strain without *HMX1* gene knockout were set as the control group.

### 2.5 Complementation experiment for LA-tolerant or sensitive knockout strains

From the molecular barcoded yeast (MoBY) open reading frame (ORF) library, Escherichia coli strains harboring complementary plasmids containing shared tolerant or sensitive knockout genes were carefully selected. Following extensive cultivation, the plasmids were extracted and subsequently transformed into knockout strains on SD-Ura agar plates for rigorous screening. A spot assay was conducted to systematically compare the growth patterns of the non-knockout strain, the complemented strain, and the knockout strain on YPD+G418 solid medium, including medium supplemented with 100 mM LA. Photographic documentation was utilized to capture the growth performance, and a thorough analysis of these images was performed to validate the tolerance of the knockout gene in Saccharomyces cerevisiae to levulinic acid. The comprehensive results of this complementation experiment are presented in Supplementary Figure S8.

### 2.6 Transcriptome analysis

Total RNA was extracted using the TRIzol reagent (Thermo Fisher, batch number 15,596,018) and then assessed for quantity and integrity using the RNA Nano 6000 Assay Kit on the Bioanalyzer 2100 system (Agilent Technologies, California, USA). Gene expression levels were estimated using FPKM (Fragments Per Kilobase of transcript per Million mapped reads). Differential expression analysis was performed using the DESeq2 R package. The criterion for significant differential expression was set as |log2 (foldchange)| ≥ 1 (padj ≤ 0.05). KEGG and GO enrichment analysis of differentially expressed genes (DEGs) was performed using the ClueGo program in Cytoscape. All parameters used in the RNA-seq experiments are presented in Supplementary material 1.

### 2.7 Metabolome analysis

In non-targeted metabolome analysis, UHPLC-MS/MS analysis was performed on the samples using a Vanquish UHPLC system (ThermoFisher, Germany) coupled with an Orbitrap Q Exactive™ HF mass spectrometer (Thermo Fisher, Germany). Raw data files were processed using Compound Discoverer 3.1 (CD3.1, ThermoFisher) to perform peak alignment, peak detection, and quantitative analysis of each metabolite. Statistical analysis was performed using statistical software R and Python. These metabolites were annotated using the KEGG database.

### 2.8 Integrated analysis of transcriptome and metabolome

A network diagram can visually represent the relationship between metabolites and genes. Selected top 10 differentially expressed genes and top 5 differentially expressed metabolites were used for plotting. All differentially expressed genes and metabolites obtained were mapped to the KEGG pathway database to obtain their common pathway information, determining the major biochemical and signal transduction pathways that both differentially expressed metabolites and genes participate in. iPath (interactive Pathways Explorer [https://pathways.embl.de/]) is an online analysis tool for visualizing metabolic pathways. It summarizes various metabolic pathways in biological systems, with nodes representing various biochemical molecules and lines representing biochemical reactions. The enriched pathways shared by both differentially expressed metabolites and genes were mapped to the iPath website. Based on the above enrichment results, a metabolic pathway map was presented for the metabolic and transcriptomic enrichment.

## 3 Results and analysis

### 3.1 Screening of key genes and spot plating experiment verification

**Figure 1:**
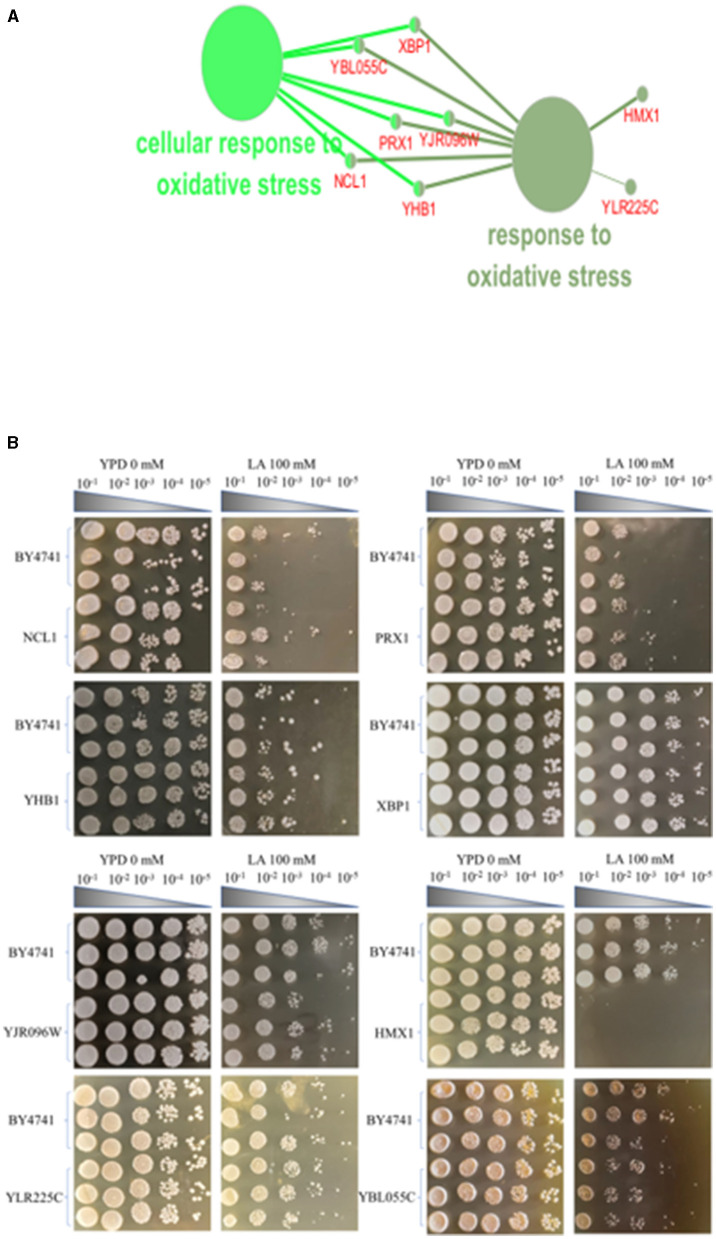
Screening of knockout strains sensitive to levulinic acid (LA) in *S. cerevisiae*. **(A)** KEGG and GO enrichment analysis of genes sensitive to levulinic acid (LA) in Saccharomyces cerevisiae. **(B)** Spot test of strain BY4741 on YPD medium with varying concentrations of LA. Spot test validation of the key knockout strain of *Saccharomyces cerevisiae* in YPD medium containing 100 mM LA.

More than 4,400 gene knockout strains of Saccharomyces cerevisiae were inoculated on solid plates containing LA using a large-scale cell manipulation platform. Solid plate photography, SGAtool software scanning analysis, and weight value ≥0.2 screening were conducted. KEGG and GO enrichment analysis of differential phenotypic genes using Cytoscape software revealed that the genes *HMX1, YLR225C, XBP1, YBL055C, YJR096W, PRX1, NCL1*, and *YHB1* on the membrane fluidity pathway exhibited sensitivity to LA (as shown in Supplementary Table S1). The selected weight values were 0.33, 0.63, 0.30, 0.41, 0.30, 0.40, 0.36, and 0.32, respectively. Figure 1A illustrates the eight key genes enriched on the membrane fluidity pathway. The genes *XBP1, YBL055C, YJR096W, PRX1, NCL1*, and *YHB1* are all associated with cellular oxidative stress responses, while *HMX1* and *YLR225C* are only related to oxidative stress.

In the screening experiment of knockout strains for the eight key genes in the oxidative stress pathway—*HMX1, YLR225C, XBP1, YBL055C, YJR096W, PRX1, NCL1*, and *YHB1*—these strains formed colony spot at dilution of 10^−4^ and colony spot at dilution of 10^−5^ colonies on solid media containing YPD+G418, displaying consistent growth patterns with the BY4741 standard strain. On YPD+G418 solid media containing 100 mM LA, both the BY4741 standard strain and the NCL1 and *PRX1* knockout strains formed colony spot at dilution of 10^−2^, showing no significant differences in growth. The knockout strains of *YHB1, YJR096, YLR225C*, and *YBL055C* also formed colony spot at dilution of 10^−3^ and colony spot at dilution of 10^−4^ under the same conditions, exhibiting consistent growth patterns with the BY4741 standard strain. The *XBP1* knockout strain showed no significant difference in growth compared to the non-knockout BY4741 strain, both forming colony spot at dilution of 10^−5^. Notably, on YPD+G418 solid media with 100 mM LA, the BY4741 standard strain formed colony spot at dilution of 10^−3^ and colony spot at dilution of 10^−4^, while the *HMX1* knockout strain failed to form any colonies, indicating a significant difference in growth compared to the BY4741 standard strain (Figure 1B). In this experiment, dot blot tests revealed that seven of the knockout strains (*YLR225C, XBP1, YBL055C, YJR096W, PRX1, NCL1*, and *YHB1*) showed no significant difference in LA tolerance compared to the non-knockout BY4741 strain. However, the tolerance to LA significantly weakened after knocking out the *HMX1* gene, suggesting that this gene plays a role in promoting the tolerance of Saccharomyces cerevisiae under LA stress.

### 3.2 Transcriptomic and metabolomic analysis of *HMX1* knockout strain under LA stress conditions

**Figure 2:**
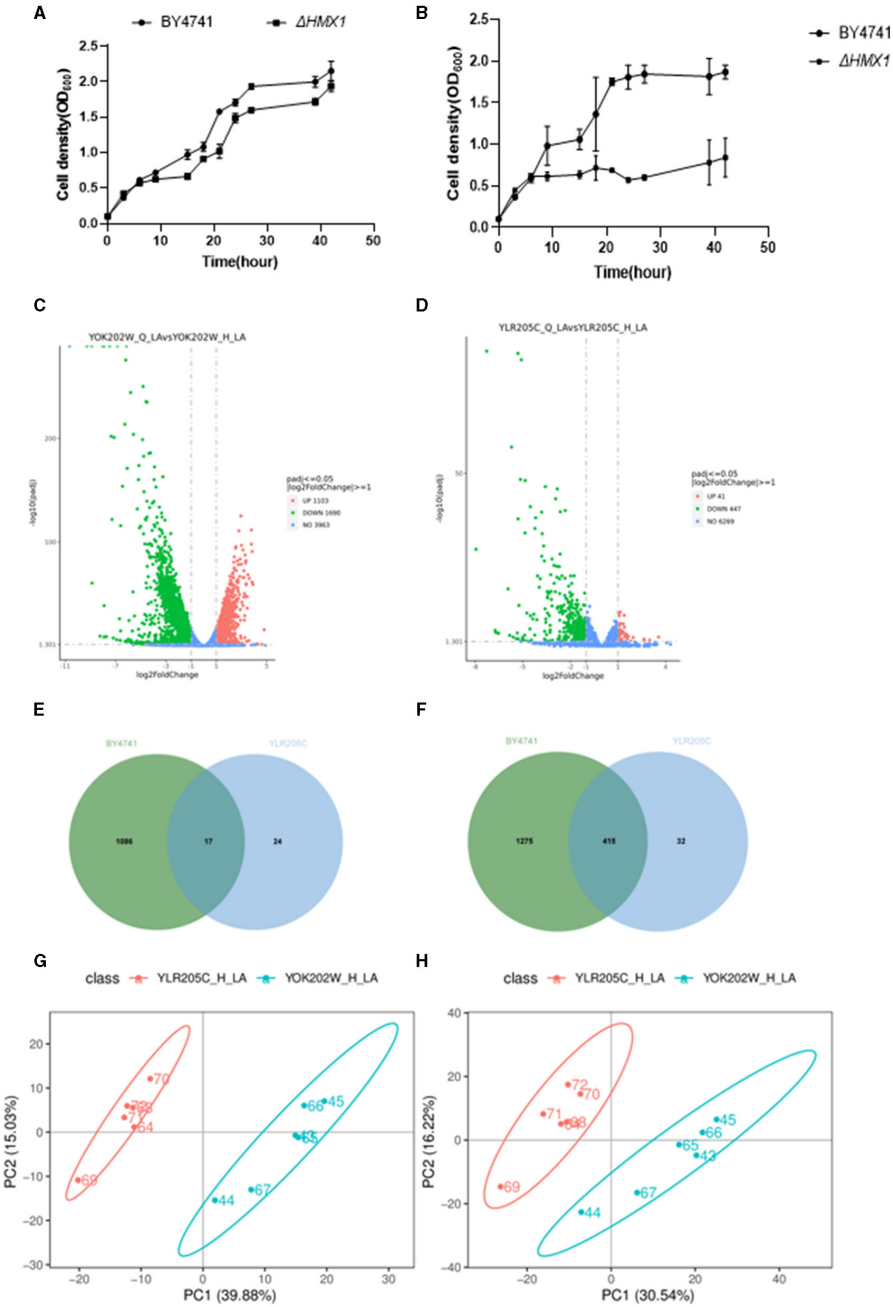
Analysis of growth, transcriptome, and metabolome of BY4741 strain and *HMX1*Δ knockout strain under 100mM levulinic acid (LA) conditions. The growth trajectory of BY4741 strain and *HMX1*Δ knockout strain in YPD medium **(A)** and YPD medium containing 100Mm LA **(B)**. Volcano plot illustrating the genes with differential expression in the BY4741 strain **(C)** and *HMX1*Δ **(D)** after LA treatment for 3 h. **(E)** Venn diagram analysis of up-regulated genes in the *HMX1*Δ strain compared to the BY4741 strain under LA stress. **(F)** Venn diagram analysis of down-regulated genes in the *HMX1*Δ strain compared to the BY4741 strain under LA stress. Principal component analysis of positively charged **(G)** and negatively charged **(H)** metabolites between strain *HMX1*Δ and BY4741.

To investigate the tolerance mechanism of *HMX1* gene in Saccharomyces cerevisiae under LA stress, this experiment conducted a differential analysis of the transcriptomic and metabolomic levels between the *HMX1* knockout strain and BY4741 under LA stress. As shown in Figure 2A, there was no significant difference in the growth rate between the *HMX1* knockout strain and the BY4741 strain in YPD+G418 media, indicating that the gene knockout did not have a significant impact on cell proliferation. However, as shown in Figure 2B, in media containing 100 mM LA, the growth rate of the *HMX1* knockout strain was significantly lower than that of the BY4741 strain, suggesting that the knockout of the *HMX1* gene significantly affected the strain's tolerance to LA. To further explore the molecular mechanism of this phenomenon, cells from the *HMX1* knockout strain and the BY4741 strain treated with LA stress for 3 h were collected and subjected to transcriptomic and metabolomic analyses.

Based on the data analysis in Supplementary Table S2, the total amount of sequencing clean bases for all samples significantly exceeded 6.0 G. Given that the Saccharomyces cerevisiae genome contains approximately 6,000 genes, with an average gene length of about 1,000 base pairs (bp), and a transcript containing approximately 6 × 10^6^ bp, it can be concluded that the sequencing depth of this experiment exceeded 1,000 × . Typically, a sequencing depth of 100 × is sufficient for analysis, but the sequencing depth of this experiment far exceeds this standard, indicating an ample amount of data. In addition, the Q20 and Q30 quality scores of the sequencing data were both above 90%, indicating a very low sequencing error rate and high data quality. In summary, this sequencing produced sufficient and high-quality data, fully meeting the needs for in-depth analysis. As shown in Supplementary Figure S1, this figure demonstrates the results of the correlation analysis between samples. The analysis revealed a high positive correlation among biologically replicated samples, validating the consistency of data obtained from each replicate experiment and providing a reliable data foundation for subsequent research.

After performing background removal analysis on the sequencing data of the BY4741 (YOK202W) standard strain and the *HMX1* (YLR205C) knockout strain, we compared the gene expression profiles before and after LA treatment. The study found that in the BY4741 reference strain, LA treatment resulted in altered expression of 2,793 genes, with 1,086 genes upregulated and 1,275 genes downregulated (Figure 2E). In contrast, in the *HMX1* knockout strain, a total of 488 genes showed altered expression, with 24 genes upregulated and 32 genes downregulated (Figure 2F). The volcano plots (Figures 2C, D) displayed the differentially expressed genes in the three strains, laying a foundation for further analysis.

A Venn diagram (Figure 2G) was used to compare the downregulated genes between the *HMX1* knockout strain and the BY4741 reference strain. It was found that the BY4741 strain had 1,275 uniquely downregulated genes, while the *HMX1* knockout strain had 32 uniquely downregulated genes. There were 1,307 downregulated genes shared between the two strains. Cytoscape software was employed to perform gene enrichment analysis on the 32 uniquely downregulated genes in the *HMX1* knockout strain. The results indicated that these genes were mainly enriched in three pathways: organic anion transport, carboxylic acid transport, and monocarboxylic acid transport.

Pearson correlation analysis (Supplementary Figures S2, S3) demonstrated a high correlation between replicate samples in the metabolomics sequencing, confirming the reliability of the data and providing a solid foundation for subsequent analysis. Principal component analysis of the metabolomic profiles after LA treatment (Figures 2G, H) revealed significant differences between the *HMX1* knockout strain and the BY4741 reference strain, indicating differences in their metabolite composition.

Under stress conditions, the differential expression of metabolites in each strain was analyzed. It was found that there were 71 and 111 metabolites upregulated and downregulated in the *HMX1* knockout strain, respectively, while 83 and 95 metabolites were upregulated and downregulated in the BY4741 strain, respectively. Venn diagram analysis showed that there were 144 and 197 metabolites that were commonly upregulated and downregulated in both the *HMX1* knockout strain and the BY4741 strain (Supplementary Figures S4, S5). To gain a deeper understanding of the specific expression of metabolites in the *HMX1* knockout strain, a metabolite enrichment analysis was conducted. The results showed that the specifically upregulated metabolites in the *HMX1* knockout strain were enriched in pathways such as Fructose and mannose metabolism, Purine metabolism, and Pyrimidine metabolism (Supplementary Figure S6). The specifically downregulated metabolites in the *HMX1* knockout strain were enriched in pathways such as Fatty acid biosynthesis, Riboflavin metabolism, and Pantothenate and COA biosynthesis (Supplementary Figure S7).

### 3.3 Knockout of *HMX1* affects the formation of cell wall-membrane system

**Figure 3:**
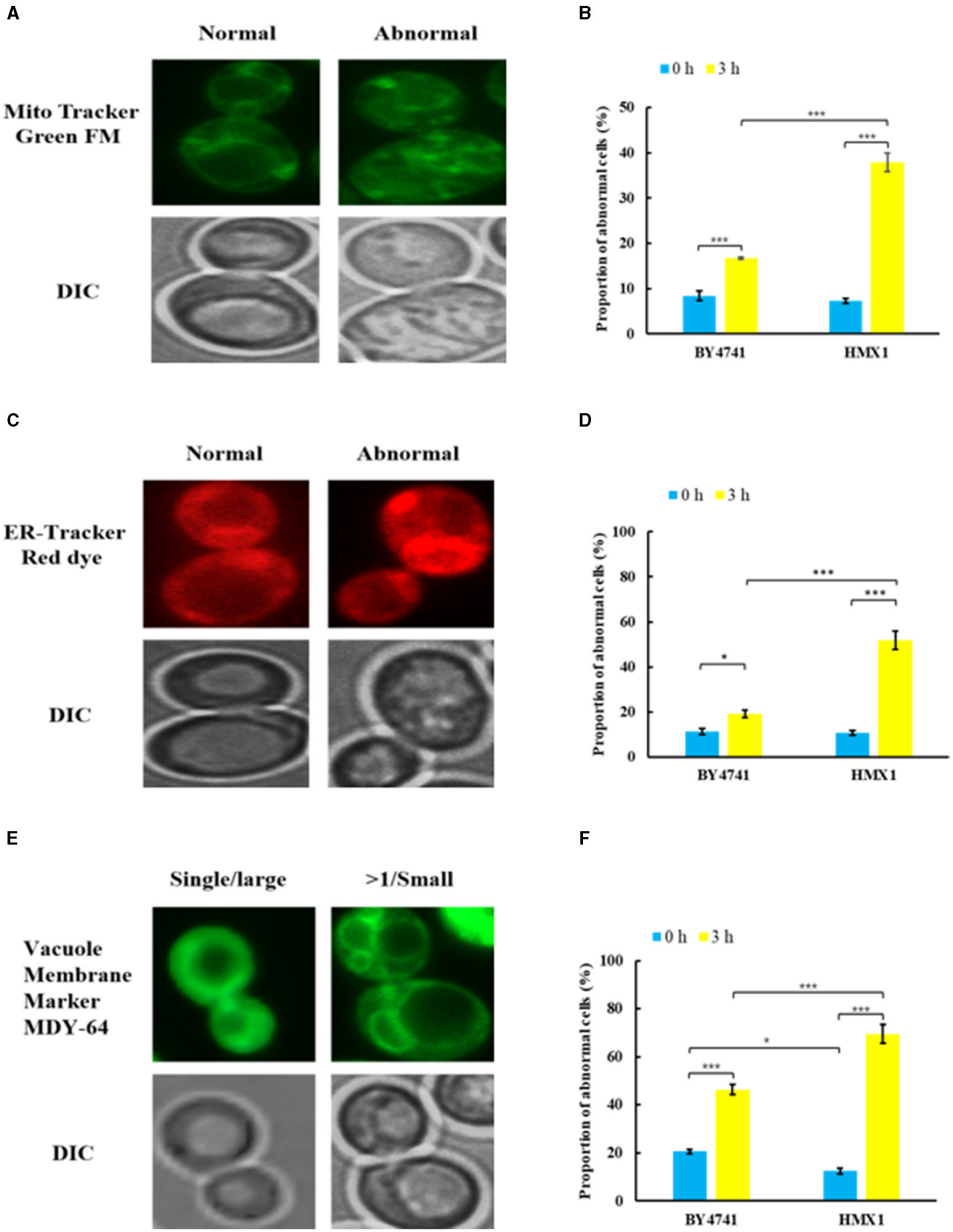
Morphological changes of mitochondria (MT), endoplasmic reticulum (ER), and vacuoles (VC) in the strain BY4741 and *HMX1*Δ under LA stress. Different morphologies of MT **(A)**, ER **(C)**, VC **(E)** in cells. The proportion of cells that displayed abnormal MT **(B)**, abnormal ER **(D)**, and more than single small VC **(F)** after LA treatment for 0 and 3 h. Mito Tracker Green FM: the mitochondria-specific dye. ER-Tracker Red dye: Endoplasmic reticulum stain. Vacuole Membrane Marker MDY-64: vacuole dyeing agent. Single/large: single large vacuole. >1/ Small: more than single small vacuole. DIC, differential interference microscope. *p < 0.05, ***p < 0.001 indicates significant differences. The data represents averages of three experiments. At least 100 cells were examined on each bright-field image.

To clarify the effect of *HMX1* knockout on the membrane system, this experiment observed the endoplasmic reticulum, mitochondria, and vacuoles, which have membrane structures (Figure 3). As shown in Figures 3A, C, under LA stress, both mitochondria and the endoplasmic reticulum exhibited different degrees of damage. From Figure 3B, it can be seen that after 3 h of treatment, the proportion of cells with mitochondrial damage in the BY4741 reference strain increased from 6.73% to 16.77%, while the proportion of cells with mitochondrial damage in the *HMX1* knockout strain increased from 7.26% to 37.95%. Additionally, after 3 h of treatment, the proportion of cells with endoplasmic reticulum damage in the BY4741 reference strain increased from 11.30% to 19.21%, while the proportion of cells with endoplasmic reticulum damage in the *HMX1* knockout strain increased from 10.75% to 51.86%. Regarding vacuole damage, the proportion of cells with vacuole damage in the BY4741 reference strain increased from 20.59% to 46.34%, while the proportion of cells with vacuole damage in the *HMX1* knockout strain increased from 11.76% to 69.43%. From the above results, it can be seen that under LA stress, the mitochondria, endoplasmic reticulum, and vacuoles with membrane structures in the *HMX1* knockout strain exhibited significant damage.

**Figure 4:**
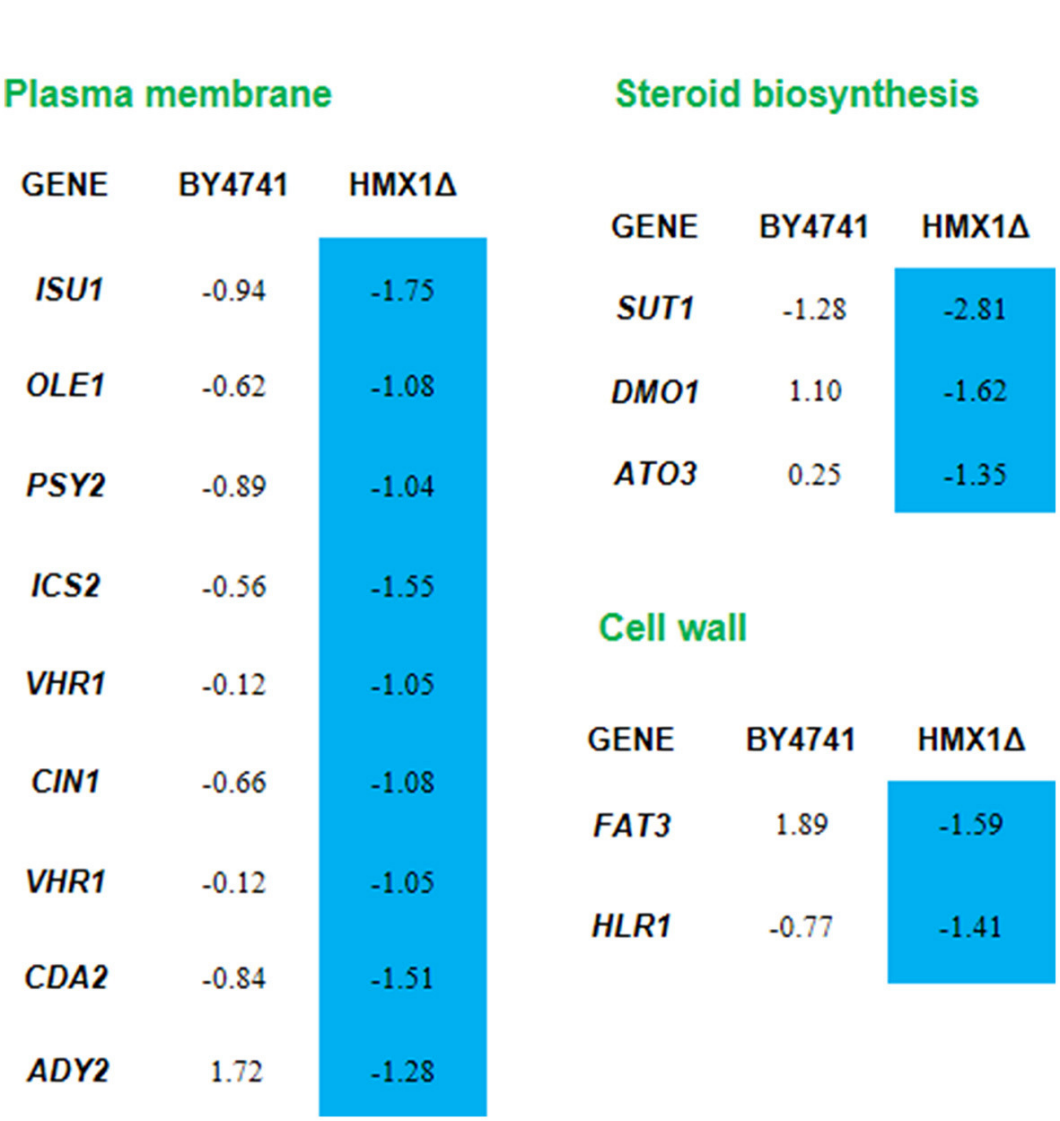
Response mechanism of the cell wall-membrane system to levulinic acid (LA) in the *HMX1*Δ strain. Expression levels of genes in the BY4741 and *HMX1*Δ strains in response to 100 mM LA, represented by the values of log2(fold change). Blue indicates significant downregulation of the gene.

Analysis of transcriptome sequencing data revealed that genes specifically downregulated in the *HMX1* knockout strain were enriched in the monocarboxylic acid transport pathway (Supplementary Table S3). These results confirm, at the gene expression level, that the knockout of *HMX1* caused damage to the membrane system. Through the enrichment and collation of downregulated genes, it was found that the genes ADY2, ATO3, and FAT3 responsible for organic anion transport and carboxylic acid transport were specifically downregulated by 1.28, 1.35, and 1.59 times, respectively, in the *HMX1* knockout strain (Figure 4). All these genes are related to the energy metabolism and cell membrane of Saccharomyces cerevisiae cells. In addition, since *HMX1* is closely related to sterol synthesis, this experiment also investigated other genes related to cell wall system synthesis, such as the *HLR1* gene, which showed significant differences in the *HMX1* knockout strain (Figure 4). As can be seen from Figure 4, nine genes related to the plasma membrane were downregulated to some extent in the *HMX1* knockout strain, while the downregulation was much less pronounced in the BY4741 strain. Overall, the knockout of *HMX1* is likely to result in insufficient iron accumulation, which inhibits sterol acquisition, thereby reducing the stability of the cell wall and weakening the resistance of the membrane system to LA.

### 3.4 Knockout of *HMX1* inhibits coenzyme-A synthesis

**Figure 5:**
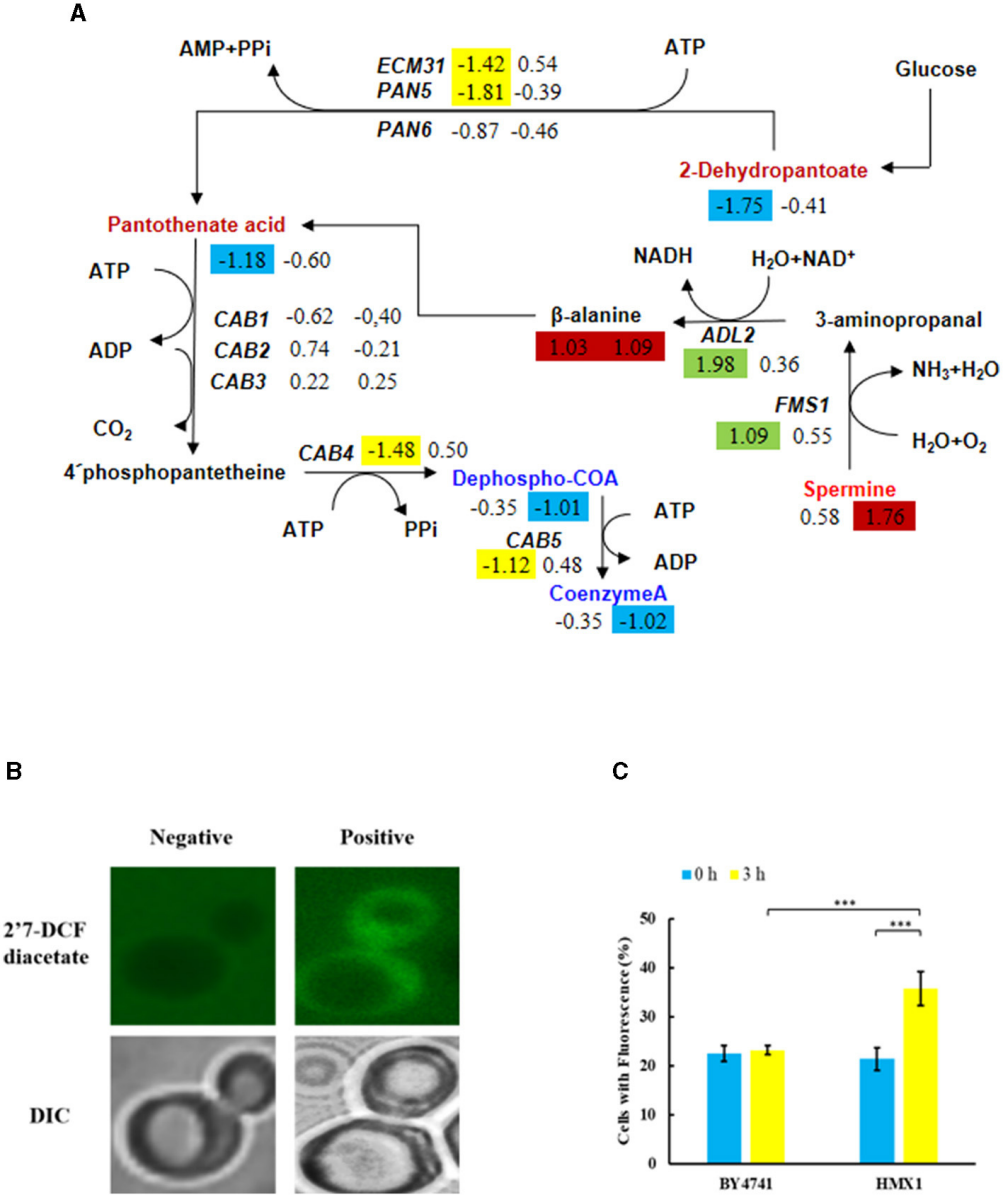
Under LA stress, the decrease in pantothenate-mediated Acetyl-CoA (CoA) in BY4741 and *HMX1*Δ strains leads to an increase in reactive oxygen species (ROS). **(A)** Changes in the expression levels of key genes and the contents of key metabolites in the pantothenate synthesis and metabolism pathway. The metabolite contents and gene expression levels in BY4741 strain (left column) and *HMX1*Δ strain (right column) after 3 h of treatment with 100 mM LA compared to 0 h treatment are represented by log2 (fold change) values. Green indicates significant upregulation of genes. Yellow indicates significant downregulation of genes. Red indicates a significant increase in metabolite content. Blue indicates a significant decrease in metabolite content. **(B)** Accumulation of ROS in cells. Changes in ROS content **(C)** and necrotic cell ratio in BY4741 and *HMX1*Δ strains after 0 and 3 h of LA treatment. 2′7′-Dichlorodihydrofluorescein diacetate: ROS indicator dye. DAPI, DNA-specific dye diamidino-2-phenylindole. ***p < 0.001 indicates a significant difference. Data represent the mean of three experiments. At least 100 cells were examined per bright-field image.

Metabolomic analysis of the *HMX1* knockout strain and BY4741 strain exposed to LA revealed that specifically downregulated metabolites in the *HMX1* knockout strain were enriched in the Pantothenate and COA biosynthesis pathway. In the *HMX1* knockout strain, metabolites related to pantothenate and COA synthesis, such as Dephospho-COA and Coenzyme-A, exhibited specific downregulation by 1.01 and 1.02 times, respectively, whereas in the BY4741 strain, they were downregulated by only 0.35 times. Additionally, the precursor of Coenzyme-A, Pantothenate, underwent a specific downregulation of 1.18 times in the BY4741 strain, which shifted to a specific downregulation of only 0.60 times in the *HMX1* knockout strain. These findings suggest that the knockout of the *HMX1* gene has a certain degree of influence on the biosynthesis of pantothenate and COA (Figure 5A). Simultaneously, it was observed that there were no significant differences in the expression levels of genes *CAB1, CAB2*, and *CAB3* related to the synthesis of these metabolites in both the *HMX1* knockout strain and BY4741 strain under LA stress. However, *CAB4* and *CAB5* exhibited specific downregulation by 1.48 and 1.12 times, respectively. This indicates that the changes in Dephospho-COA and related metabolites are associated with changes in gene transcription levels. Intracellularly, Dephospho-COA reduces oxidative stress by transferring acetyl groups, protecting cells from damage caused by free radicals. As shown in Figures 5B, C, the proportion of cells with reactive oxygen species (ROS) in the BY4741 strain was 22.48% and 23.15% at 0 h and 3 h after LA treatment, respectively, indicating that LA does not stimulate the accumulation of intracellular ROS in BY4741 cells. However, in the *HMX1* knockout strain, the proportion of cells with ROS increased significantly from 21.37% to 35.73% after 3 h of LA treatment, suggesting that under LA stress and with the absence of the *HMX1* gene, the accumulation of ROS is significantly elevated. This demonstrates that the efficiency of ROS clearance is significantly reduced in the *HMX1* knockout strain, which is likely related to the decrease in Coenzyme-A (Figure 5A). Previous studies have shown that other precursors of acetyl-CoA, such as pyruvate or acetate, can enter other intracellular compartments, resulting in reduced availability of acetyl-CoA in the cytoplasm (Schadeweg and Boles, 2016). Our experimental findings revealed that while the content of Pantothenate, a precursor for Coenzyme-A synthesis, showed a certain degree of accumulation, the content of Coenzyme-A in the *HMX1* knockout strain under LA stress increased by 1.02 times compared to that in the BY4741 strain under LA stress. We speculate that the knockout of the *HMX1* gene under LA stress leads to reduced Coenzyme-A synthesis.

### 3.5 Knockout of *HMX1* impacts riboflavin metabolism

**Figure 6:**
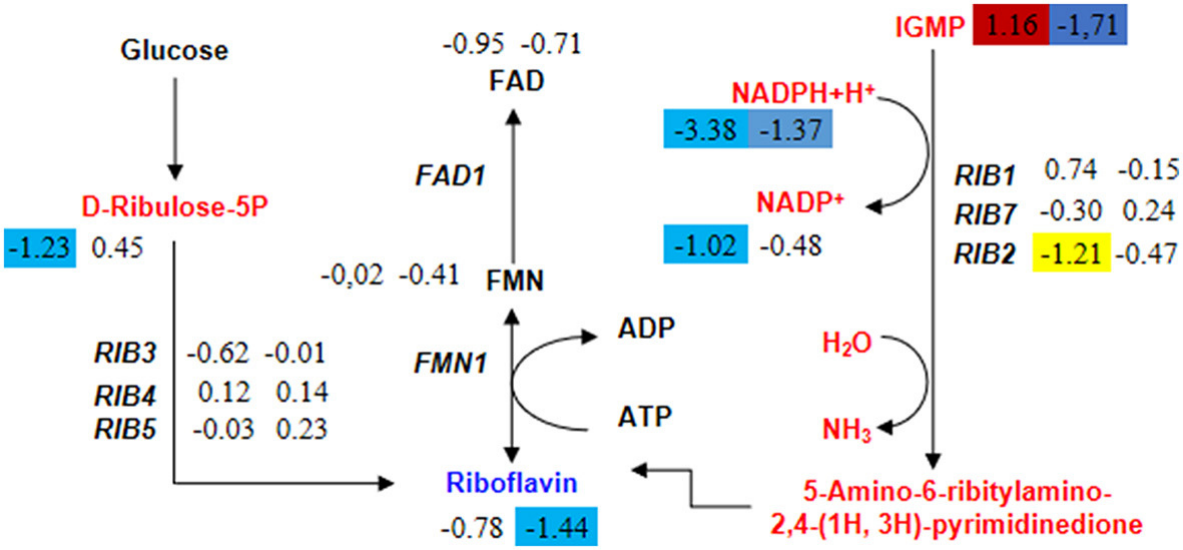
Changes in the expression levels of key genes and the content of key metabolites in the riboflavin metabolism pathway in BY4741 and *HMX1*Δ strains after 100 mM LA treatment. The metabolite content and gene expression levels [represented by log2 (fold change) values] in BY4741 strain **(left column)** and *HMX1*Δ strain **(right column)** after 3 h of treatment with 100 mM LA, compared to the treatment for 0 h. Yellow indicates significant downregulation of genes. Red indicates a significant increase in metabolite content. Blue indicates a significant decrease in metabolite content.

Acetyl-CoA is an essential cofactor involved in various metabolic pathways. As mentioned earlier, the knockout of the *HMX1* gene hinders the synthesis of acetyl-CoA. Therefore, this experiment found that the specifically downregulated metabolites in the *HMX1* knockout strain were also enriched in the Riboflavin metabolism pathway. Riboflavin is synthesized through enzymatic reactions involving GTP and ribulose-5-phosphate catalyzed by enzymes encoded by the riboflavin biosynthetic operon. We speculate that the specific downregulation of key metabolites in this pathway may affect riboflavin synthesis. In the riboflavin metabolism, the levels of Riboflavin were significantly reduced by 1.44 times in the *HMX1* knockout strain under LA stress. However, there was no specific increase or decrease in the BY4741 strain under LA stress. Notably, the precursor of Riboflavin, IGMP, exhibited a significant increase of 1.16 times in the BY4741 strain under LA stress, while it decreased by 1.71 times in the *HMX1* knockout strain under the same conditions (Figure 6). Additionally, the expression levels of the key enzyme genes involved in this pathway, namely *RIB1, RIB3, RIB4, RIB5*, and *RIB7*, exhibited similar trends in both strains under LA stress (Figure 6). This suggests that the transcriptional levels of these key enzyme genes do not affect the increase or decrease in the levels of key metabolites in the Riboflavin metabolism pathway. Based on the above results, it can be concluded that the decrease in IGMP is a crucial factor leading to the reduction in Riboflavin content. Taken together, we infer that under LA stress and with the knockout of the *HMX1* gene, the synthesis of riboflavin is reduced.

## 4 Conclusion and discussion

In this experiment, we confirmed through spot plate assays that the oxidative stress pathway plays an important role in LA tolerance. Additionally, the gene *HMX1* identified in this study has not been previously studied in the context of LA tolerance, but we found that it can enhance the tolerance of Saccharomyces cerevisiae to levulinic acid, suggesting that it is a new gene in the oxidative stress pathway involved in LA tolerance. Through subcellular structural observations, we found that this gene has protective effects on mitochondria, endoplasmic reticulum, vacuoles, and reactive oxygen species.

According to the analysis of omics, the knockout of *HMX1* has caused downregulation of genes related to the cell wall and cell membrane system under LA stress. However, there was no significant downregulation of membrane system and cell wall-related genes in the *HMX1* knockout strain without LA stress. The above results indicate that the knockout of *HMX1* affects the expression level of related genes, rather than being caused by LA. Therefore, it can be inferred that *HMX1* has a protective effect on the cell membrane. The knockout of the *HMX1* gene may weaken the ability of Saccharomyces cerevisiae to cope with oxidative stress, resulting in an increase in the level of intracellular reactive oxygen species (ROS), and thus increasing the possibility of cell damage (Collinson et al., 2011). *HMX1* is a homolog of heme oxygenase, which plays a role in oxidative stress response, heme catabolism, and intracellular iron homeostasis. It has been found to be localized on the endoplasmic reticulum and outer nuclear membrane (Protchenko and Philpott, 2003). Yeast cells regulate sterol uptake through heme, where sterols are critical components of cell membranes (Jordá and Puig, 2020). Previous studies have found that the deletion of *HMX1* leads to iron accumulation defects and decreased heme degradation activity in yeast cells, resulting in the accumulation of heme in yeast cells (Protchenko and Philpott, 2003). Moreover, some studies have reported that the growth of Saccharomyces cerevisiae under iron-deficient conditions requires the synthesis of ergosterol (Jordá et al., 2021). Therefore, it can be speculated that the iron accumulation defects caused by the knockout of the *HMX1* gene inhibit the uptake of sterols by BY4741. The effects of weak acids on Saccharomyces cerevisiae are usually considered to be due to the accumulation of protons or acid ions in the cytoplasm, leading to acidification, which may have toxic effects on the normal metabolic functions of cells (Russell, 1992; Ullah et al., 2012). Therefore, cells need more energy and resources to deal with the reconstruction and maintenance of the cell wall, and the deletion of *HMX1* may lead to the blockage of these processes, resulting in unstable exchange of substances inside and outside the cell. Knockout of the *HMX1* gene may further exacerbate this situation, as *HMX1* may be involved in regulating gene expression or protein function related to cell membrane permeability. Ergosterol molecules are transported in the form of sterol esters to multiple subcellular structures, such as mitochondria, vacuoles, and the plasma membrane and inner membrane systems of the endoplasmic reticulum (Girardi Piva et al., 2022). In this study, under LA stress, HMX1 knockout strains exhibited damage to their mitochondria, vacuoles, and endoplasmic reticulum (Figure 2). Consequently, it is plausible to deduce that the ergosterol content, which is indirectly regulated by HMX1 in these knockout strains, undergoes a reduction. This reduction, in turn, weakens the cell membrane-wall system, thereby compromising the resistance of Saccharomyces cerevisiae to LA.

Research results indicate that overexpression of the *OLE1* gene leads to increased oleic acid content and increased unsaturation of fatty acids on the plasma membrane, which in turn enhances the tolerance of cells to acetoin (Guo et al., 2018).

However, this study found that nine genes related to the cell membrane, including *ISU1* and *OLE1*, showed specific downregulation in the *HMX1* knockout strain under LA stress, leading to cell membrane damage and reduced tolerance to LA. The cholesterol pathway, which is also included, plays a crucial role in membrane stability. Since the key gene *SUT1* responsible for the cholesterol pathway shows specific downregulation in the *HMX1* knockout strain under LA stress. Other synthesis-related genes such as DMO1 and ATO3 also show specific downregulation in both strains under LA stress. Therefore, it can be speculated that the cholesterol content may decrease in the *HMX1* knockout under LA stress. As cholesterol is a key component for maintaining the integrity and adaptability of the cell membrane, increasing the level of cholesterol helps enhance the stability and resistance of the cell membrane (Vázquez et al., 2019). Therefore, it can be inferred that the knockout of the *HMX1* gene may interfere with the production of ergosterol and cholesterol, indicating that *HMX1* plays an important role in maintaining the protective function of the cell membrane and enhancing the tolerance of yeast to LA.

This experiment found that under LA stress, the active oxygen content did not accumulate significantly in the non-knockout strain, indicating that LA does not lead to the accumulation of active oxygen in the BY4741 standard strain cells. However, the active oxygen content increased in the *HMX1* knockout strain under LA stress. Previous studies have found that the genes involved in oxygen radical detoxification in Saccharomyces cerevisiae (*GRX2, HMX1, SOD1, TRX3*, and *TSA1*) include the knockout gene *HMX1* (Olzhausen et al., 2009). Therefore, it can be speculated that the increase in active oxygen content is caused by the knockout of *HMX1*. However, it may also be related to acetyl-CoA, which exists in the cytoplasm and mitochondria of Saccharomyces cerevisiae cells. The synthesis of acetyl-CoA starts with pantothenic acid in Saccharomyces cerevisiae. Previous studies have found that the pantothenate kinase reaction limits the synthesis of CoA in yeast and is inhibited by acetyl-CoA (Vadali et al., 2004; Philpott and Protchenko, 2008). Within the cell, Dephospho-CoA reduces its own oxidative stress by transferring acetyl groups and protecting itself from free radical damage. We speculate that under LA stress, the knockout strain *HMX1* allows Saccharomyces cerevisiae to quickly adjust its internal metabolism based on growth conditions. The relatively small increase in pantothenic acid in the *HMX1* knockout strain under LA stress compared to the non-knockout strain *HMX1* under LA stress may be a compensatory mechanism for the cell to cope with the absence of the *HMX1* gene. However, the increased content is not sufficient to support the synthesis of Coenzyme-A, leading to an increase in intracellular active oxygen. This suggests that the efficiency of active oxygen scavenging is significantly reduced in the *HMX1* gene knockout strain, which is likely related to the decrease in Coenzyme-A.

Additionally, this study found that the riboflavin content also decreased in the *HMX1* knockout strain. Riboflavin is an essential compound for yeast growth. The production of riboflavin is significantly influenced by the supply of its precursor substances, and increasing the supply of these precursors will help improve the yield of riboflavin (You et al., 2021). Therefore, it can be inferred that the decrease in riboflavin content may be directly related to the reduction in the content of precursor substances. The trigger for overproduction of riboflavin in yeast is associated with the activation of intracellular stress signaling cascades in response to environmental changes. Related studies have shown that the results of riboflavin overproduction caused by oxidative stress in *A. gossypii* are related to mycelial growth and stress defense against ROS (Kato et al., 2020). In addition, iron-limiting conditions induce the overproduction of riboflavin in various yeasts, such as Candida guilliermondii, Candida flava, and Candida famata (Prokopiv et al., 2013; Andreieva et al., 2020). As mentioned earlier, the deletion of the *HMX1* gene leads to defects in iron accumulation, and iron deficiency can lead to excessive synthesis of riboflavin in several yeast species. Therefore, it can be speculated that the stress mechanism that prevents riboflavin from defending against ROS accumulation is impeded in the *HMX1* knockout strain under LA stress. Furthermore, the contents of NADPH and NADP+ in the *HMX1* knockout strain under LA stress showed a certain increase compared to the non-knockout strain BY4741 under LA stress. Riboflavin has an antioxidant effect on oxidative stress, especially lipid peroxidation. The mechanism of riboflavin protecting the body from oxidative stress can be attributed to the glutathione redox cycle. Moreover, research results indicate that riboflavin functions as a precursor of the coenzymes flavin adenine dinucleotide (FAD) and flavin mononucleotide (FMN) to alter cell metabolism and increase the NADH/NADPH ratio, thereby enhancing resistance to oxidative stress (Olfat et al., 2022). Overall, the increase in riboflavin content may improve the tolerance of the *HMX1* knockout strain to LA stress.

The results of this study have revealed the response mechanism of Saccharomyces cerevisiae *HMX1* knockout strain in response to LA stress. The study found that increasing the content of cell membrane-wall system, pantothenic acid, acetyl-CoA, and riboflavin is crucial for enhancing the resistance of Saccharomyces cerevisiae to LA. These findings provide a scientific basis for the modification of yeast strains that are tolerant to LA, and also open up new possibilities for the use of LA as a raw material for fermentation to produce high-value-added products. This lays a theoretical foundation for modifying strains to improve their tolerance to levulinic acid, which is of great significance for the efficient conversion of lignocellulosic biomass resources into levulinic acid.

## Data availability statement

The data presented in the study are deposited in the NCBI repository, accession number PRJNA1088400.

## Author contributions

JT: Validation, Software, Project administration, Methodology, Investigation, Formal analysis, Data curation, Conceptualization, Writing – original draft. YC: Validation, Software, Project administration, Methodology, Investigation, Data curation, Conceptualization, Writing – original draft. QL: Supervision, Software, Methodology, Investigation, Conceptualization, Writing – original draft, Validation, Data curation. WX: Supervision, Project administration, Investigation, Data curation, Conceptualization, Writing – original draft. XX: Writing – original draft, Validation, Data curation, Conceptualization. XC: Writing – original draft, Validation, Formal analysis, Data curation. LixY: Writing – original draft, Software, Formal analysis, Investigation. BM: Writing – original draft, Supervision, Software, Investigation, Validation. JL: Writing – original draft, Validation, Supervision, Project administration, Methodology, Formal analysis, Software. FL: Writing – original draft, Methodology, Software, Formal analysis. CF: Writing – original draft, Validation, Methodology, Supervision. WLo: Writing – original draft, Methodology, Data curation, Project administration, Formal analysis. HL: Writing – original draft, Software, Methodology, Data curation, Formal analysis. XH: Writing – original draft, Methodology, Data curation, Supervision, Software. PF: Writing – original draft, Validation, Methodology, Supervision, Project administration. WLi: Writing – original draft, Software, Methodology, Data curation, Project administration, Formal analysis. KZ: Writing – original draft, Software, Resources, Project administration. LiuY: Writing – original draft, Visualization, Resources, Formal analysis. YY: Visualization, Resources, Project administration, Writing – review & editing. MM: Visualization, Resources, Project administration, Writing – review & editing. HW: Visualization, Resources, Project administration, Formal analysis, Writing – review & editing.
