# Supplementary material for: The response mechanism analysis of HMX1 knockout strain to levulinic acid in Saccharomyces cerevisiae
Source: Front Microbiol. 2024 Jun 26;15:1416903. doi: 10.3389/fmicb.2024.1416903 (PMC11233763; doi:10.3389/fmicb.2024.1416903)
Supplement: Supplementary file 1 [file Data_Sheet_1.zip › Supplementary Image 8.pdf]

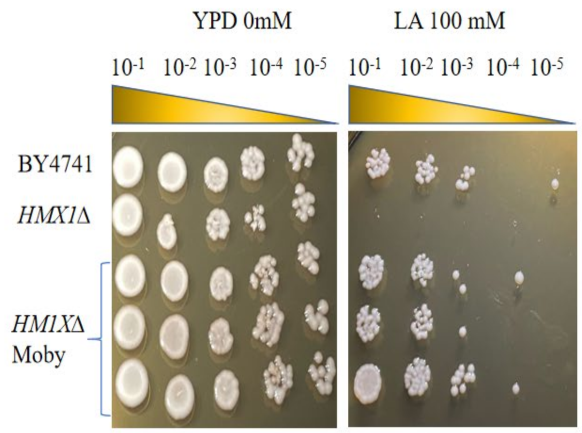

Figure S8. Dot-blot validation results of the Moby complemented strain *HMX1Δ* represents the strain of BY4741 with the *HMX1* gene knocked out.

Through the observation of the complemented strain (Figure S1), it is found that the *HMX1Δ*+ Moby complemented strain forms colonies at the dilution of 10^{-4} in the environment of levulinic acid, proving that the complementation effect is good. In addition, compared with the knockout strain, the complemented strain still has significant growth recovery, indicating that the *HMX1* gene plays an important role in the tolerance to levulinic acid.
