# Supplementary material for: The response mechanism analysis of HMX1 knockout strain to levulinic acid in Saccharomyces cerevisiae
Source: Front Microbiol. 2024 Jun 26;15:1416903. doi: 10.3389/fmicb.2024.1416903 (PMC11233763; doi:10.3389/fmicb.2024.1416903)
Supplement: Supplementary file 1 [file Data_Sheet_1.zip › Supplementary Tables 1-3 and Figures 1-7.pdf]

Table S1 The key gene involved in the sensitivity of the membrane fluidity pathway to levulinic acid

AN	AO	NC size (EX)	NC std. (EX)	NC std.dev (CN)	Score	Score std	p-Value
NCL1	*YBL024W*	1.60	0.04	0.00	0.63	0.04	0.00
PRX1	*YBL064C*	1.30	0.03	0.02	0.30	0.03	0.00
YHB1	*YGR234W*	1.46	0.04	0.00	0.40	0.04	0.00
XBP1	*YIL101C*	1.36	0.05	0.02	0.41	0.05	0.00
YJR096W	*YJR096W*	1.37	0.03	0.02	0.36	0.03	0.00
HMX1	*YLR205C*	1.33	0.01	0.04	0.33	0.01	0.00
YLR225C	*YLR225C*	1.38	0.05	0.05	0.32	0.05	0.00
YBL005C	*YBL005C*	1.34	0.21	0.03	0.30	0.21	0.00

注（Note）: AN: Array Name; AO: Arry ORF; NC Size: Normalized colony size; NC std: Normalized colony std.dev

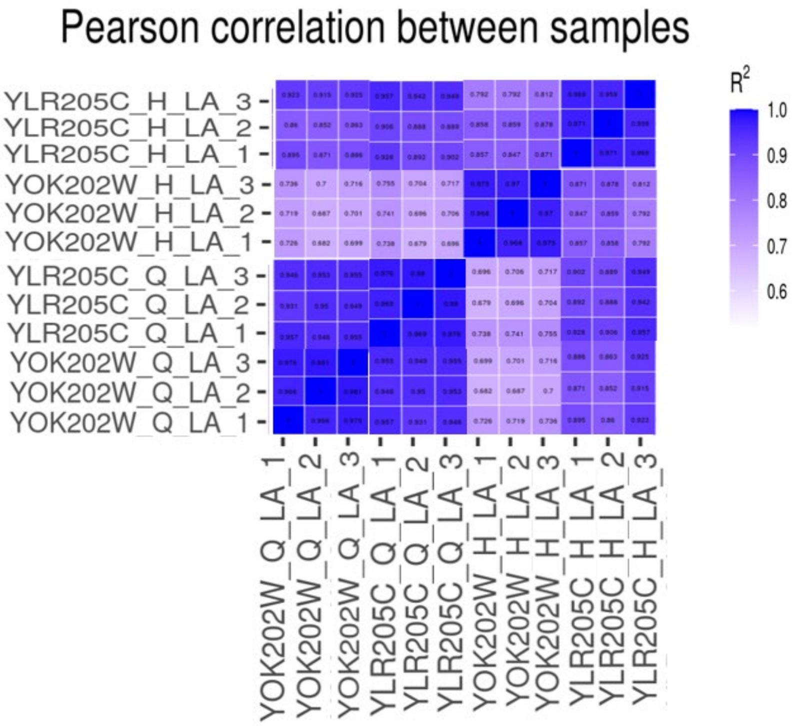

Figure S1 Pearson correlation between transcriptome sequencing data of the strain BY4741 and *HMX1*Δ. YOK202W_Q_LA and YOK202W_H_LA represent the strain BY4741 treated with LA for 0 h and 3 h, respectively. YLR205C_Q_LA and YLR205C_H_LA represent the strain *HMX1*Δ treated with LA for 0 h and 3 h, respectively.

Table S2. The summary statistics table for sequencing data.

Sample	Clean reads (M)	Clean bases (G)	Q20	Q30	GC Content	Sequencing depth (×)
YOK202W_Q_LA_1	40,846,138	6.13	97.3	92.32	41.42	1021
YOK202W_Q_LA_2	40,773,696	6.12	97.31	92.32	41.46	1033
YOK202W_Q_LA_3	46,036,434	6.91	97.52	92.88	41.3	1152
YLR205C_Q_LA_1	39,850,022	5.98	97.34	92.52	41.37	997
YLR205C_Q_LA_2	45,684,368	6.85	97.32	92.47	41.25	1142
YLR205C_Q_LA_3	41,892,994	6.28	97.23	92.21	41.27	1046
YOK202W_H_LA_1	40,050,578	6.01	97.3	92.38	40.92	1001
YOK202W_H_LA_2	42,000,640	6.3	97.52	92.86	40.88	1050
YOK202W_H_LA_3	41,151,488	6.17	97.55	92.95	40.99	1028
YLR205C_H_LA_1	41,320,440	6.2	97.48	92.78	41.23	1033
YLR205C_H_LA_2	41,015,978	6.15	97.5	92.87	41.02	1025
YLR205C_H_LA_3	40,872,182	6.13	97.51	92.86	41.26	1022

Sequencing depth=Clean bases/(Total number of genes×gene length). Total number of all the genes is about 6000 in *Saccharomyces cerevisiaes* (https://www.ncbi.nlm.nih.gov/genome), and average length of all the genes in *S. cerevisiae*is estimated to 1000 bp.

Table S3. An analysis focusing on the enrichment of specifically downregulated genes in the *HMX1*Δ strain.

ID	Term	Associated Genes Found
GO:0015711	organic anion transport	*ADY2*, *ATO3*, *FAT3*
GO:0046942	carboxylic acid transport	*ADY2, ATO*3, *FAT3*
GO:0015718	monocarboxylic acid transport	*ADY2*, *ATO3*, *FAT3*

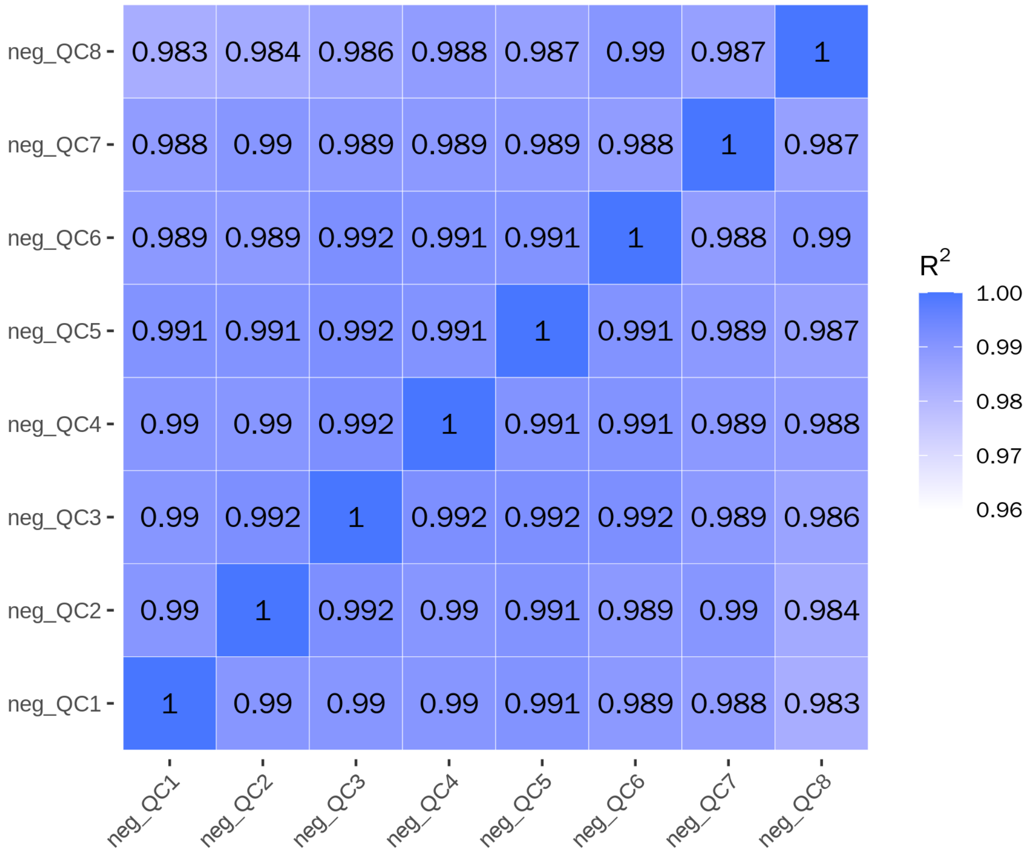

Figure S2 Pearson correlation analysis of positive metabolites between replicate samples.

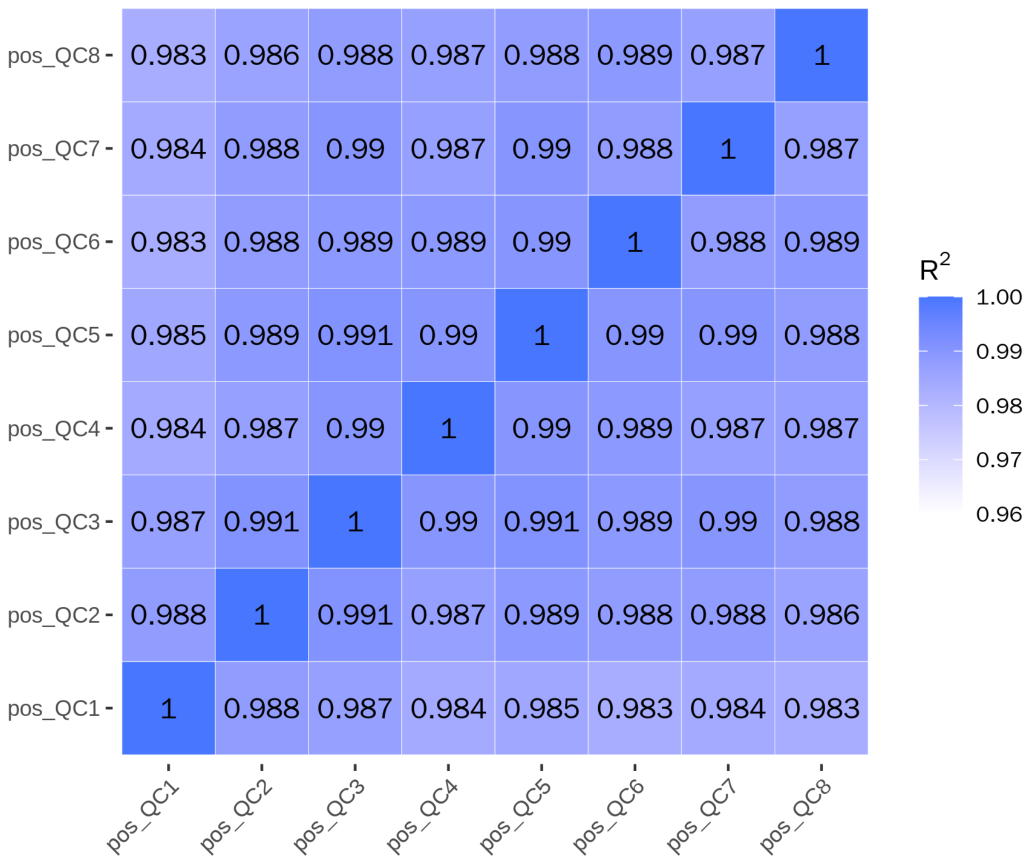

Figure S3 Pearson correlation analysis of negative metabolites between replicate samples.

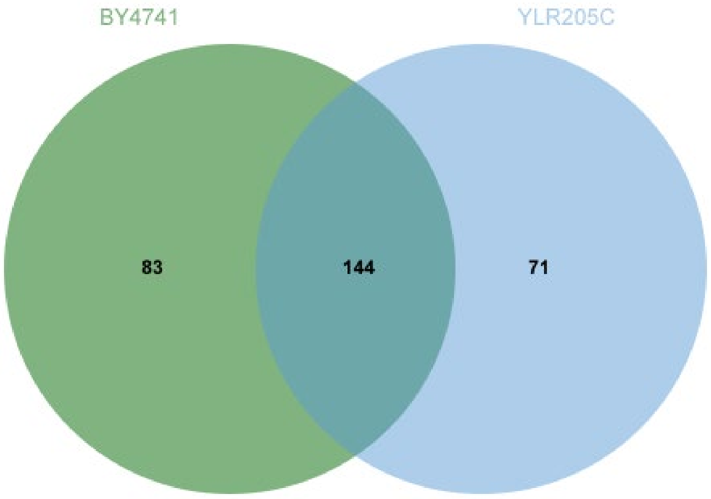

Figure S4 Venn diagram of the up-regulated metabolites in the strain BY4741 and *HMX1*Δ. YOK202W represent the up-regulated metabolites in the strain BY4741 after LA treatment for 3 h. YLR205C represent the up-regulated metabolites in the strain *HMX1*Δ after LA treatment for 3 h.

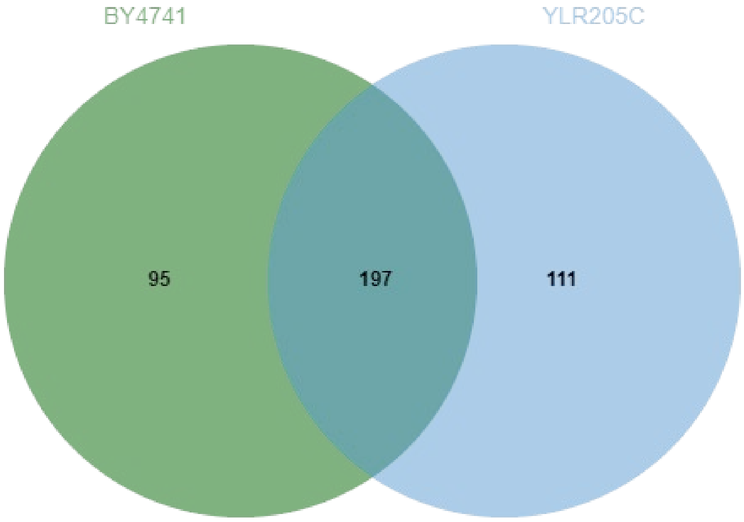

Figure S5 Venn diagram of the down-regulated metabolites in the strain BY4741 and *HMX1*Δ. YOK202W represent the down-regulated metabolites in the strain BY4741 after LA treatment for 3 h. **YLR205C** represent the down-regulated metabolites in the strain *HMX1*Δ after LA treatment for 3 h.

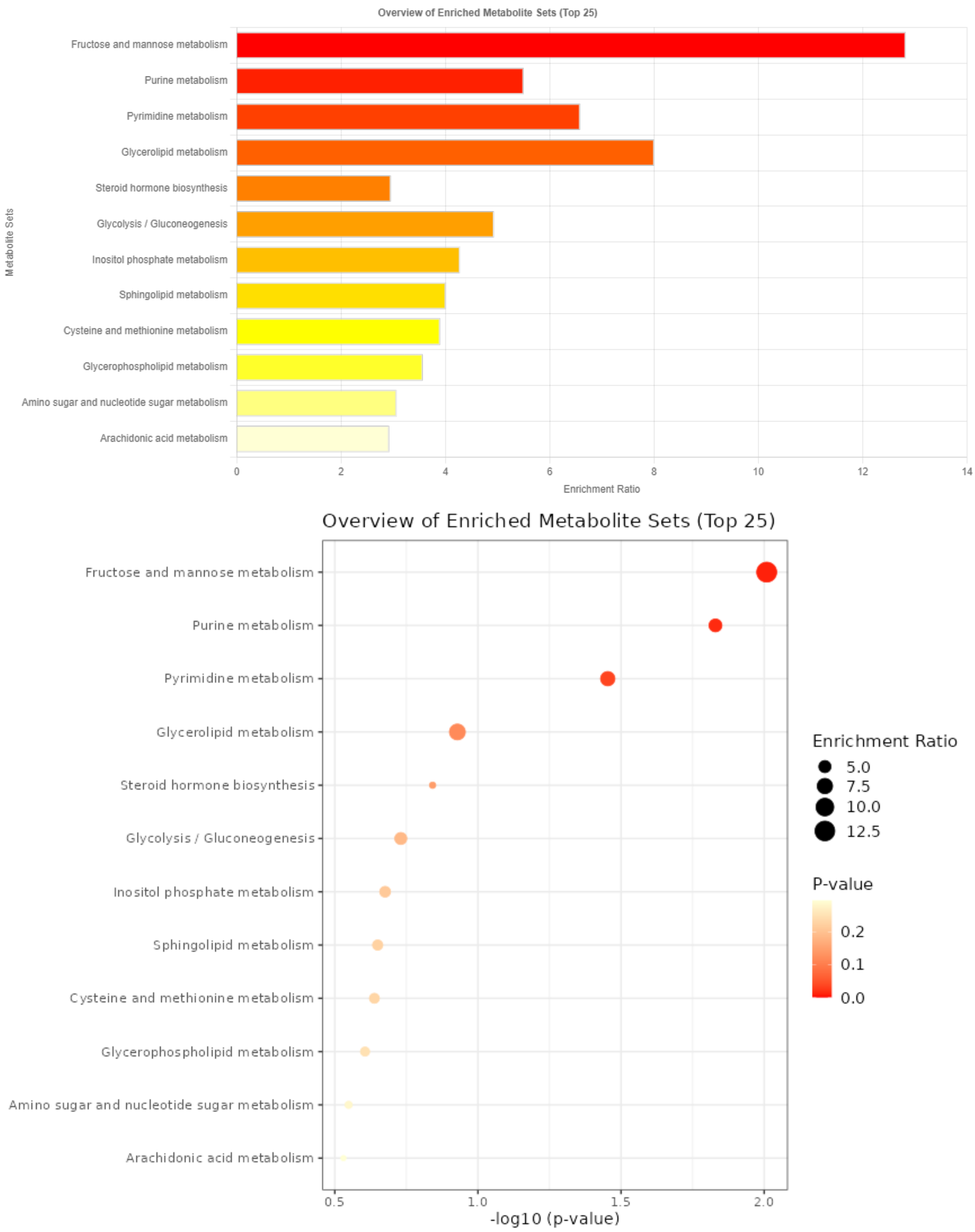

Figure S6 Enrichment analysis of specific metabolites up-regulated in the strain *HMX1*Δ.

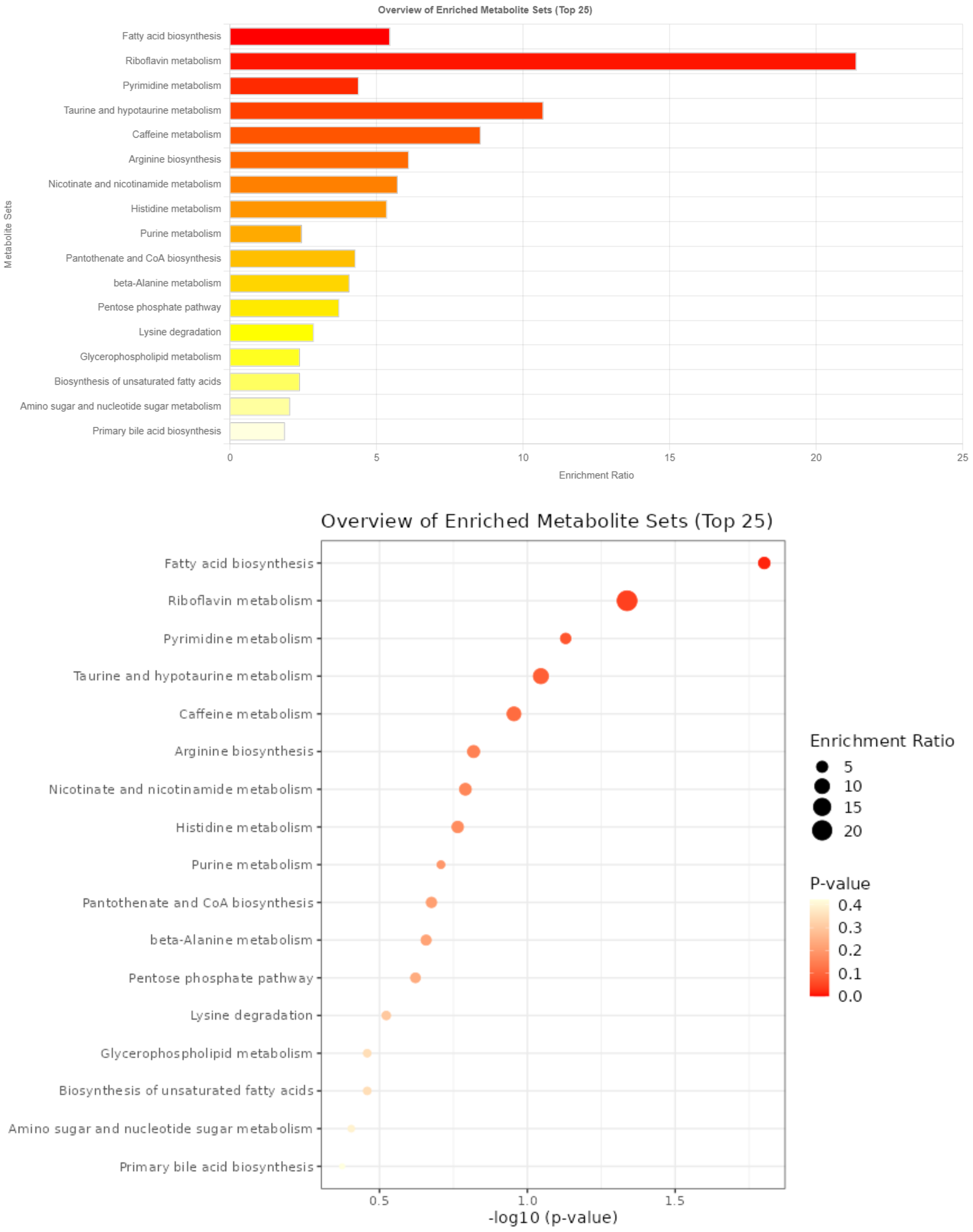

Figure S7 Enrichment analysis of specific metabolites down-regulated in the strain *HMX1*Δ.
