# Supplementary material for: The response mechanism analysis of HMX1 knockout strain to levulinic acid in Saccharomyces cerevisiae
Source: Front Microbiol. 2024 Jun 26;15:1416903. doi: 10.3389/fmicb.2024.1416903 (PMC11233763; doi:10.3389/fmicb.2024.1416903)
Supplement: Supplementary file 2 [file Table_1.DOCX]

**Table S1.** Primers used for qRT-PCR of the 21 selected genes

| Number | Gene | Primer ID | Forward primer (5'-3') |
| --- | --- | --- | --- |
| 1 | OAR1 | qOAR1_F | CCGCCCTTGAAACAGAAATA |
|  |  | qOAR1_R | AGCCTGCGCAGTTAATCAAT |
| 2 | ETR1 | qETR1_F | ACCGAGTTATCCCATTGCAG |
|  |  | qETR1_R | CAGATACAGTTGCCGCAGAA |
| 3 | HMG1 | qHMG1_F | CCATCAACTGGATCGAAGGT |
|  |  | qHMG1_R | AACTCAACCAATGCGGAAAC |
| 4 | ERG8 | qERG8_F | ACAGCTTTGGCCTCCTTTTT |
|  |  | qERG8_R | CGCTTCCAATTTTACCCTGA |
| 5 | PUT1 | qPUT1_F | CAACGGCAATAGTGTGATGG |
|  |  | qPUT1_R | AAGCTGTTCAAGGTCGCAAT |
| 6 | NTH1 | qNTH1_F | AAGAGCTTTCCAAGCAAGCA |
|  |  | qNTH1_R | CCGAGACCGTTAGGATGGTA |
| 7 | ATH1 | qATH1_F | CTACGGGACCTTGTGTCGAT |
|  |  | qATH1_R | CTCGTCATCAATCCCTTCGT |
| 8 | TPS2 | qTPS2_F | CAGCAGTCCTACTGCCAACA |
|  |  | qTPS2_R | GTAATGCTGGACGGGAGAAA |
| 9 | UGP1 | qUGP1_F | CCACATGATCGAGACTGGTG |
|  |  | qUGP1_R | TTGGGCGACTTCCAATAAAC |
| 10 | GSY1 | qGSY1_F | TGATGTGGACCAAGAAGCTG |
|  |  | qGSY1_R | GCAGTGATTTGCGACACAGT |
| 11 | TRP4 | qTRP4_F | TCTGGCATATTGATCCGACA |
|  |  | qTRP4_R | CTTTAGGGCCGTATGAAGCA |
| 12 | GPD1 | qGPD1_F | ACCCGACAATTTGGTTGCTA |
|  |  | qGPD1_R | GCTACAGATACGGGGCAAAA |
| 13 | HOR2 | qHOR2_F | GGTGCAGTTAAGCTGTGCAA |
|  |  | qHOR2_R | CCAGATGCTCGAACCATTTT |
| 14 | GUT1 | qGUT1_F | ACCGATTGCATCTACGGTTC |
|  |  | qGUT1_R | ATTATGGTGGCTCTGGCATC |
| 15 | PDB1 | qPDB1_F | CCAGACTGCCAACATCATTG |
|  |  | qPDB1_R | TCTGACGGTCATCGTCTTTG |
| 16 | CDC19 | qCDC19_F | TGATGATGTTGAAGCCCGTA |
|  |  | qCDC19_R | CCTTGAAACCTTGGATGGAA |
| 17 | ACS1 | qACS1_F | TGATGACGCGCTAAGAGAGA |
|  |  | qACS1_R | CTGTTGCCCAATCCAAATCT |
| 18 | ALD3 | qALD3_F | CCAGGGCTGCTTTTGATAAC |
|  |  | qALD3_R | TCTCTAATGCGGCAAGTGTG |
| 19 | PDR10 | qPDR10_F | CTCGGGTTTGAATCAAGGAA |
|  |  | qPDR10_R | AAAAGGGAAGATTGGCTCGT |
| 20 | SNQ2 | qSNQ2_F | TATCAAAAGCTGGCCAATCC |
|  |  | qSNQ2_R | GTTTGTCCACCCTTCCTCAA |
| 21 | HFA1 | qHFA1_F | CTGGGTCGCCTATGTTTGTT |
|  |  | qHFA1_R | CCGCCTTTGTATGGAACAGT |


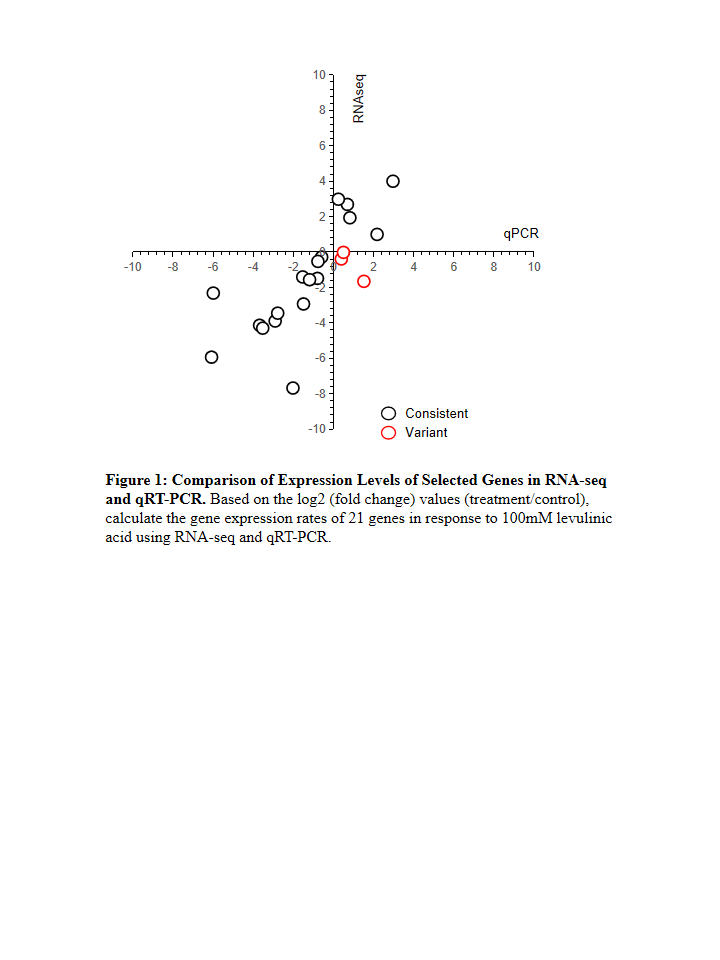


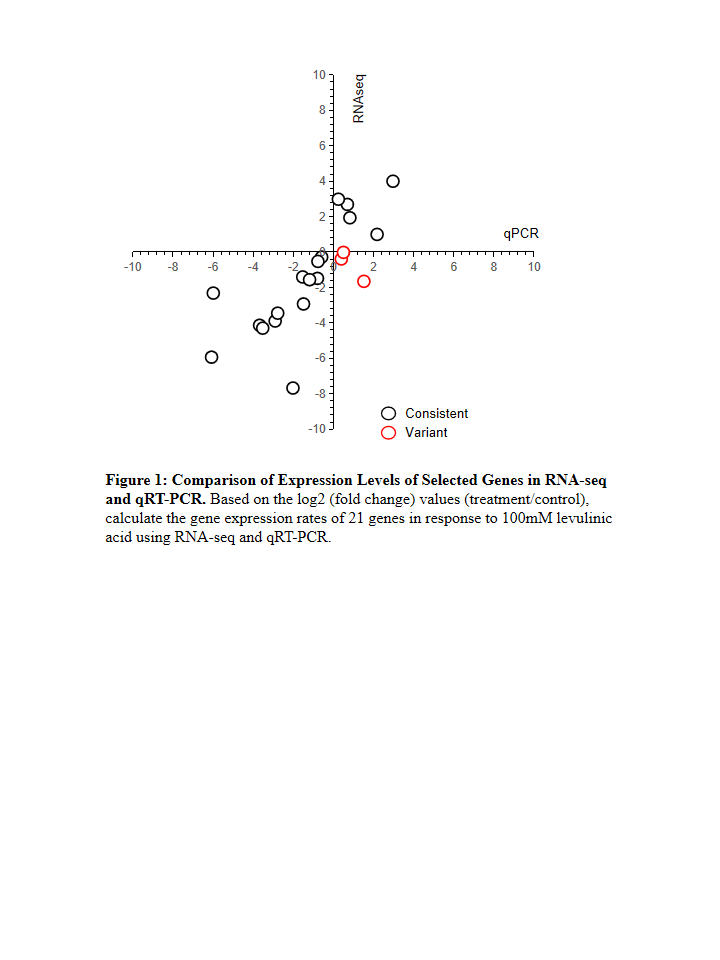


**Figure S1. Comparison of Expression Levels of Selected Genes in RNA-seq and qRT-PCR**. Based on the log2 (fold change) values (treatment/control), calculate the gene expression rates of 21 genes in response to 100mM levulinic acid using RNA-seq and qRT-PCR.
