# Supplementary material for: The response mechanism analysis of HMX1 knockout strain to levulinic acid in Saccharomyces cerevisiae
Source: Front Microbiol. 2024 Jun 26;15:1416903. doi: 10.3389/fmicb.2024.1416903 (PMC11233763; doi:10.3389/fmicb.2024.1416903)
Supplement: Supplementary file 3 [file Table_2.DOCX]

**Table S1.** SGAtool analyzed the scores of each knockout strain under *HMF* conditions

| Array ORF | Array Name | Normalize d colony size (EXPERI MENT) | Normalized colony std. dev. (EXPERIM ENT) | Normaliz ed colony size (CONTR  OL) | Normaliz ed colony std. dev. (CONTR  OL) | Score | Sco re stde v | p- Valu e |
| --- | --- | --- | --- | --- | --- | --- | --- | --- |

| YML076C | WAR1 | 0 | 0 | 1.06452 | 0.021 | -1.06918 | 0 | #NUM! |
| --- | --- | --- | --- | --- | --- | --- | --- | --- |
| YPL058C | PDR12 | 0 | 0 | 1.01884 | 0.001 | -1.0191 | 0 | #NUM! |
| YOR270C | VPH1 | 0.03359 | 0.014 | 0.999 | 0.001 | -0.96577 | 0.014 | 0.01676 |
| YMR015C | ERG5 | 0.06444 | 0.019 | 0.99054 | 0.031 | -0.93573 | 0.019 | 0.00652 |
| YOR267C | HRK1 | 0.12053 | 0.01 | 1.03893 | 0.005 | -0.91754 | 0.01 | 0.00014 |
| YGL148W | ARO2 | 0.00608 | 0.011 | 0.83432 | 0.013 | -0.83512 | 0.011 | 0.42265 |
| YNR005C | YNR005C | 0.30026 | 0.026 | 1.09235 | 0.055 | -0.78773 | 0.026 | 0.00018 |
| YML008C | ERG6 | 0 | 0 | 0.76662 | 0.02 | -0.7677 | 0 | #NUM! |
| YOL006C | TOP1 | 0.035 | 0.014 | 0.79577 | 0.03 | -0.76702 | 0.014 | 0.01574 |
| YNR006W | VPS27 | 0.32073 | 0.024 | 1.05693 | 0.034 | -0.73204 | 0.024 | 0.00011 |
| YNL097C | PHO23 | 0.10051 | 0.033 | 0.81704 | 0.034 | -0.70821 | 0.033 | 0.00863 |
| YKL081W | TEF4 | 0.28733 | 0.04 | 0.99679 | 0.015 | -0.7072 | 0.04 | 0.00073 |
| YFL025C | BST1 | 0.21606 | 0.022 | 0.91418 | 0.029 | -0.70198 | 0.022 | 0.00029 |
| YBR095C | RXT2 | 0.27082 | 0.037 | 0.96649 | 0.012 | -0.69448 | 0.037 | 0.0007 |
| YBR164C | ARL1 | 0.317 | 0.032 | 0.99801 | 0.019 | -0.68237 | 0.032 | 0.00028 |
| YEL031W | SPF1 | 0.25781 | 0.019 | 0.92967 | 0.034 | -0.67694 | 0.019 | 0.00011 |
| YDR431W | YDR431W | 0.32617 | 0.017 | 0.97787 | 0.012 | -0.65145 | 0.017 | 0.00004 |
| YBR178W | YBR178W | 0.38876 | 0.013 | 1.02386 | 0.002 | -0.63191 | 0.013 | 0.00001 |
| YJL175W | YJL175W | 0.37587 | 0.029 | 1.00362 | 0.006 | -0.62877 | 0.029 | 0.00013 |
| YBR095C | RXT2 | 0.24933 | 0.011 | 0.87182 | 0.003 | -0.6211 | 0.011 | 0.00067 |
| YNR047W | FPK1 | 0.37846 | 0.014 | 1.00345 | 0.032 | -0.61876 | 0.014 | 0.00001 |
| YDR159W | SAC3 | 0.15292 | 0.029 | 0.77109 | 0.041 | -0.61141 | 0.029 | 0.00181 |
| YLR261C | YLR261C | 0.49063 | 0.017 | 1.0931 | 0.004 | -0.6015 | 0.017 | 0.00001 |
| YCR034W | FEN1 | 0.03079 | 0.032 | 0.62304 | 0.017 | -0.59654 | 0.032 | 0.15122 |
| YMR123W | PKR1 | 0.39204 | 0.001 | 0.98478 | 0.011 | -0.59047 | 0.001 | 0 |
| YDR486C | VPS60 | 0.55668 | 0.036 | 1.14659 | 0.019 | -0.59022 | 0.036 | 0.00008 |
| YMR202W | ERG2 | 0.20738 | 0.021 | 0.7914 | 0.013 | -0.58428 | 0.021 | 0.00029 |
| YGL054C | ERV14 | 0.45326 | 0.011 | 1.03108 | 0.009 | -0.57945 | 0.011 | 0.00021 |
| YLR039C | RIC1 | 0.42853 | 0.038 | 0.99119 | 0.024 | -0.56831 | 0.038 | 0.00019 |
| YLR452C | SST2 | 0.66054 | 0.012 | 1.22542 | 0.016 | -0.56708 | 0.012 | 0 |
| YJR075W | HOC1 | 0.656 | 0.02 | 1.21455 | 0.019 | -0.56188 | 0.02 | 0.00001 |
| YGL066W | SGF73 | 0.25536 | 0.025 | 0.81596 | 0.015 | -0.5614 | 0.025 | 0.00027 |
| YBL094C | YBL094C | 0.41741 | 0.043 | 0.96201 | 0.071 | -0.54594 | 0.043 | 0.0003 |
| YLR414C | PUN1 | 0.13235 | 0.013 | 0.65965 | 0.036 | -0.52543 | 0.013 | 0.00025 |
| YER090W | TRP2 | 0.50057 | 0.015 | 1.0222 | 0.01 | -0.52018 | 0.015 | 0.00029 |
| YPR173C | VPS4 | 0.57847 | 0.009 | 1.09401 | 0.008 | -0.51393 | 0.009 | 0.00008 |
| YAL013W | DEP1 | 0.28083 | 0.018 | 0.79597 | 0.017 | -0.51067 | 0.018 | 0.00007 |
| YAL026C | DRS2 | 0.29963 | 0.039 | 0.8098 | 0.01 | -0.5086 | 0.039 | 0.00058 |
| YPR123C | YPR123C | 0.42273 | 0.017 | 0.92756 | 0.019 | -0.50591 | 0.017 | 0.00002 |
| YJR040W | GEF1 | 0.52864 | 0.085 | 1.03406 | 0.019 | -0.50384 | 0.085 | 0.00111 |
| YOR132W | VPS17 | 0.52852 | 0.019 | 1.0275 | 0.01 | -0.50164 | 0.019 | 0.00001 |
| YLR330W | CHS5 | 0.6604 | 0.043 | 1.16385 | 0.035 | -0.49936 | 0.043 | 0.00008 |
| YJL124C | LSM1 | 0.38565 | 0.004 | 0.87794 | 0.025 | -0.4941 | 0.004 | 0.00004 |
| YGL173C | XRN1 | 0.40192 | 0.007 | 0.89413 | 0.002 | -0.49191 | 0.007 | 0 |
| YOR220W | RCN2 | 0.53628 | 0.002 | 1.02751 | 0.013 | -0.488 | 0.002 | 0 |
| YBR290W | BSD2 | 0.55524 | 0.035 | 1.03803 | 0.021 | -0.48425 | 0.035 | 0.00007 |
| YLR372W | SUR4 | 0.29705 | 0.153 | 0.78687 | 0.077 | -0.48335 | 0.153 | 0.03015 |
| YGL035C | MIG1 | 0.54875 | 0.007 | 1.03178 | 0.02 | -0.47964 | 0.007 | 0.00005 |
| YPL176C | TRE1 | 0.56636 | 0.005 | 1.04912 | 0.018 | -0.47819 | 0.005 | 0.00003 |
| YDR074W | TPS2 | 0.45281 | 0.005 | 0.92836 | 0.019 | -0.47214 | 0.005 | 0.00005 |
| YLR217W | YLR217W | 0.86352 | 0.078 | 1.34835 | 0.034 | -0.47196 | 0.078 | 0.0002 |
| YDL078C | MDH3 | 0.73318 | 0.018 | 1.20628 | 0.036 | -0.47145 | 0.018 | 0.0002 |
| YEL008W | YEL008W | 0.56067 | 0.015 | 1.0246 | 0.021 | -0.46642 | 0.015 | 0.00001 |
| YAL053W | FLC2 | 0.54133 | 0.014 | 1.01103 | 0.016 | -0.46433 | 0.014 | 0 |
| YAL040C | CLN3 | 0.62035 | 0.027 | 1.07382 | 0.019 | -0.45096 | 0.027 | 0.00002 |
| YBR009C | HHF1 | 0.47322 | 0.045 | 0.91573 | 0.026 | -0.44517 | 0.045 | 0.00023 |
| YBR182C | SMP1 | 0.53806 | 0.036 | 0.98532 | 0.009 | -0.4444 | 0.036 | 0.00008 |
| YBR180W | DTR1 | 0.60385 | 0.03 | 1.04246 | 0.017 | -0.44073 | 0.03 | 0.00003 |
| YDL100C | GET3 | 0.53241 | 0.027 | 0.97029 | 0.01 | -0.43739 | 0.027 | 0.00088 |
| YBR018C | GAL7 | 0.69335 | 0.017 | 1.13951 | 0.068 | -0.43728 | 0.017 | 0.00019 |
| YKR020W | VPS51 | 0.6105 | 0.023 | 1.05275 | 0.014 | -0.43705 | 0.023 | 0.00001 |
| YDR127W | ARO1 | 0.33305 | 0.013 | 0.77302 | 0.005 | -0.43693 | 0.013 | 0.00049 |
| YDR436W | PPZ2 | 0.62542 | 0.007 | 1.06059 | 0.022 | -0.43648 | 0.007 | 0 |
| YDR474C | YDR474C | 0.62583 | 0.041 | 1.06277 | 0.011 | -0.43571 | 0.041 | 0.00008 |
| YPR160W | GPH1 | 0.50761 | 0.012 | 0.94197 | 0.012 | -0.43254 | 0.012 | 0 |
| YBL052C | SAS3 | 0.58828 | 0.02 | 1.02659 | 0.021 | -0.43172 | 0.02 | 0.00001 |
| YBR044C | TCM62 | 0.59731 | 0.02 | 1.03197 | 0.024 | -0.42781 | 0.02 | 0.00001 |
| YGR016W | YGR016W | 0.66097 | 0.001 | 1.08848 | 0.005 | -0.42723 | 0.001 | 0 |
| YNL003C | PET8 | 0.54629 | 0.048 | 0.97397 | 0.015 | -0.42662 | 0.048 | 0.00018 |
| YBR171W | SEC66 | 0.58818 | 0.045 | 1.01501 | 0.014 | -0.42126 | 0.045 | 0.00194 |
| YBL063W | KIP1 | 0.63599 | 0.016 | 1.0638 | 0.017 | -0.41889 | 0.016 | 0 |
| YNL241C | ZWF1 | 0.56777 | 0.016 | 0.98836 | 0.026 | -0.41584 | 0.016 | 0.00001 |
| YLR119W | SRN2 | 0.67869 | 0.042 | 1.09314 | 0.025 | -0.41335 | 0.042 | 0.00006 |
| YJL004C | SYS1 | 0.61743 | 0.026 | 1.03374 | 0.029 | -0.41131 | 0.026 | 0.00002 |
| YGR092W | DBF2 | 0.40823 | 0.019 | 0.81282 | 0.018 | -0.40811 | 0.019 | 0.00003 |
| YGR051C | YGR051C | 0.78284 | 0.051 | 1.19313 | 0.11 | -0.40775 | 0.051 | 0.00008 |
| YKL212W | SAC1 | 0.27896 | 0.025 | 0.68709 | 0.01 | -0.40613 | 0.025 | 0.00019 |
| YHL025W | SNF6 | 0.31419 | 0.002 | 0.7201 | 0.02 | -0.40504 | 0.002 | 0.00001 |
| YAR018C | KIN3 | 0.64434 | 0.012 | 1.04959 | 0.005 | -0.40256 | 0.012 | 0 |
| YBR184W | YBR184W | 0.57823 | 0.031 | 0.97548 | 0.03 | -0.39805 | 0.031 | 0.00004 |
| YAL058C-A | YAL058C-A | 0.56441 | 0.029 | 0.95865 | 0.015 | -0.39689 | 0.029 | 0.00004 |
| YEL017C-A | PMP2 | 0.675 | 0.053 | 1.06071 | 0.023 | -0.39196 | 0.053 | 0.00013 |
| YBL066C | SEF1 | 0.67841 | 0.004 | 1.06807 | 0.004 | -0.38681 | 0.004 | 0.00001 |
| YNL040W | YNL040W | 0.61014 | 0.015 | 1.00162 | 0.025 | -0.38655 | 0.015 | 0 |
| YNL293W | MSB3 | 0.74325 | 0.03 | 1.12991 | 0.018 | -0.38619 | 0.03 | 0.00002 |
| YBL032W | HEK2 | 0.61488 | 0.041 | 1.01222 | 0.033 | -0.38546 | 0.041 | 0.00008 |
| YBR218C | PYC2 | 0.67312 | 0.006 | 1.05874 | 0.029 | -0.3846 | 0.006 | 0 |
| YCR033W | SNT1 | 0.66013 | 0.018 | 1.04673 | 0.012 | -0.38405 | 0.018 | 0.00001 |
| YBR223C | TDP1 | 0.69158 | 0.047 | 1.0769 | 0.014 | -0.38364 | 0.047 | 0.00009 |
| YER079W | YER079W | 0.61234 | 0.084 | 0.99603 | 0.01 | -0.38267 | 0.084 | 0.00071 |
| YOR081C | TGL5 | 0.80395 | 0.011 | 1.18723 | 0.045 | -0.38264 | 0.011 | 0.00006 |
| YCL045C | EMC1 | 0.64688 | 0.013 | 1.02608 | 0.007 | -0.37988 | 0.013 | 0.00013 |
| YNL323W | LEM3 | 0.57214 | 0.035 | 0.95094 | 0.002 | -0.37974 | 0.035 | 0.00006 |
| YJR090C | GRR1 | 0.51492 | 0.022 | 0.89562 | 0.026 | -0.37967 | 0.022 | 0.00002 |
| YCL046W | YCL046W | 0.66275 | 0.015 | 1.04214 | 0.008 | -0.37567 | 0.015 | 0 |
| YER072W | VTC1 | 0.67131 | 0.042 | 1.04815 | 0.019 | -0.37516 | 0.042 | 0.00007 |
| YML035C | AMD1 | 0.55208 | 0.012 | 0.93093 | 0.04 | -0.37407 | 0.012 | 0 |
| YDL118W | YDL118W | 0.57987 | 0.022 | 0.9517 | 0.017 | -0.37098 | 0.022 | 0.00001 |
| YHR012W | VPS29 | 0.6648 | 0.009 | 1.03695 | 0.009 | -0.37088 | 0.009 | 0 |
| YBR083W | TEC1 | 0.67585 | 0.033 | 1.0451 | 0.006 | -0.36605 | 0.033 | 0.00003 |
| YPR050C | YPR050C | 0.65086 | 0.005 | 1.01707 | 0.015 | -0.36334 | 0.005 | 0.00002 |
| YJL178C | ATG27 | 0.65794 | 0.028 | 1.02275 | 0.002 | -0.36296 | 0.028 | 0.00002 |
| YCR025C | YCR025C | 0.62188 | 0.041 | 0.98591 | 0.017 | -0.36247 | 0.041 | 0.00008 |
| YIR023W | DAL81 | 0.69915 | 0.031 | 1.05566 | 0.05 | -0.36174 | 0.031 | 0.00002 |
| YER019W | ISC1 | 0.51159 | 0.033 | 0.87356 | 0.027 | -0.36116 | 0.033 | 0.00008 |
| YAL028W | FRT2 | 0.66611 | 0.018 | 1.02927 | 0.015 | -0.36058 | 0.018 | 0.00001 |
| YJL139C | YUR1 | 0.59241 | 0.05 | 0.95314 | 0.001 | -0.3594 | 0.05 | 0.00017 |
| YBL060W | YEL1 | 0.64684 | 0.021 | 1.00497 | 0.028 | -0.35916 | 0.021 | 0.00001 |
| YGR166W | TRS65 | 0.65943 | 0.019 | 1.01544 | 0.027 | -0.35788 | 0.019 | 0.00001 |
| YBR050C | REG2 | 0.69909 | 0.012 | 1.06197 | 0.023 | -0.35673 | 0.012 | 0 |
| YKL156W | RPS27A | 0.70112 | 0.008 | 1.05909 | 0.003 | -0.35636 | 0.008 | 0.00005 |
| YOR062C | YOR062C | 0.73586 | 0.024 | 1.09144 | 0.012 | -0.35431 | 0.024 | 0.00001 |
| YNL147W | LSM7 | 0.41905 | 0.005 | 0.78092 | 0.038 | -0.35246 | 0.005 | 0.00005 |
| YBR057C | MUM2 | 0.69895 | 0.015 | 1.05401 | 0.015 | -0.35161 | 0.015 | 0 |
| YIL114C | POR2 | 0.73089 | 0.014 | 1.08535 | 0.026 | -0.35149 | 0.014 | 0 |
| YDR494W | RSM28 | 0.73497 | 0.047 | 1.07576 | 0.045 | -0.35149 | 0.047 | 0.00007 |
| YOL118C | YOL118C | 0.69963 | 0.011 | 1.04826 | 0.006 | -0.35086 | 0.011 | 0 |
| YHL023C | NPR3 | 0.68392 | 0.037 | 1.03554 | 0.031 | -0.35055 | 0.037 | 0.00004 |
| YBR103W | SIF2 | 0.65637 | 0.045 | 1.00737 | 0.016 | -0.35013 | 0.045 | 0.00009 |
| YJR053W | BFA1 | 0.76012 | 0.013 | 1.10643 | 0.024 | -0.35013 | 0.013 | 0 |
| YDR451C | YHP1 | 0.70612 | 0.036 | 1.05587 | 0.022 | -0.34938 | 0.036 | 0.00004 |
| YKR055W | RHO4 | 0.40559 | 0.008 | 0.75872 | 0.029 | -0.34876 | 0.008 | 0.00013 |
| YMR276W | DSK2 | 0.69205 | 0.035 | 1.04478 | 0.04 | -0.34797 | 0.035 | 0.00004 |
| YNL198C | YNL198C | 0.76864 | 0.014 | 1.11647 | 0.004 | -0.3477 | 0.014 | 0 |
| YJL138C | TIF2 | 0.68874 | 0.046 | 1.0364 | 0.008 | -0.34607 | 0.046 | 0.00008 |
| YBR264C | YPT10 | 0.75486 | 0.035 | 1.09995 | 0.015 | -0.34605 | 0.035 | 0.00003 |
| YEL053C | MAK10 | 0.63241 | 0.024 | 0.97922 | 0.01 | -0.34556 | 0.024 | 0.00001 |
| YNL255C | GIS2 | 0.65999 | 0.057 | 1.01421 | 0.051 | -0.34549 | 0.057 | 0.00018 |
| YNL325C | FIG4 | 0.8431 | 0.034 | 1.18896 | 0.011 | -0.3454 | 0.034 | 0.00002 |
| YNL022C | YNL022C | 0.83193 | 0.013 | 1.18028 | 0.032 | -0.34525 | 0.013 | 0 |
| YBR227C | MCX1 | 0.73516 | 0.036 | 1.07842 | 0.012 | -0.34485 | 0.036 | 0.00003 |
| YCR077C | PAT1 | 0.41349 | 0.019 | 0.75805 | 0.017 | -0.34478 | 0.019 | 0.00002 |
| YPL259C | APM1 | 0.68173 | 0.02 | 1.02361 | 0.025 | -0.34397 | 0.02 | 0.00001 |
| YNL125C | ESBP6 | 0.66714 | 0.02 | 1.01105 | 0.011 | -0.34293 | 0.02 | 0.0003 |
| YBR073W | RDH54 | 0.74748 | 0.028 | 1.09053 | 0.018 | -0.34212 | 0.028 | 0.00002 |
| YIL112W | HOS4 | 0.65768 | 0.005 | 1.00129 | 0.004 | -0.34202 | 0.005 | 0 |
| YBR272C | HSM3 | 0.6908 | 0.014 | 1.03315 | 0.01 | -0.34077 | 0.014 | 0 |
| YBR034C | HMT1 | 0.64015 | 0.007 | 0.97991 | 0.017 | -0.34026 | 0.007 | 0.00004 |
| YBL068W | PRS4 | 0.71689 | 0.007 | 1.06067 | 0.021 | -0.33991 | 0.007 | 0 |
| YNR021W | YNR021W | 0.71371 | 0.022 | 1.05216 | 0.02 | -0.33917 | 0.022 | 0.00001 |
| YLR262C | YPT6 | 0.69576 | 0.016 | 1.02584 | 0.036 | -0.33893 | 0.016 | 0 |
| YPL178W | CBC2 | 0.31352 | 0.008 | 0.65406 | 0.013 | -0.33833 | 0.008 | 0.00001 |
| YAR002C-A | ERP1 | 0.71876 | 0.019 | 1.06078 | 0.003 | -0.33825 | 0.019 | 0.00001 |
| YBR042C | CST26 | 0.68451 | 0.051 | 1.02612 | 0.018 | -0.33806 | 0.051 | 0.00011 |
| YPL105C | SYH1 | 0.74918 | 0.03 | 1.08584 | 0.008 | -0.33787 | 0.03 | 0.00002 |
| YHL029C | OCA5 | 0.66923 | 0.036 | 1.00809 | 0.003 | -0.33772 | 0.036 | 0.00004 |
| YOR320C | GNT1 | 0.72447 | 0.023 | 1.04767 | 0.054 | -0.3364 | 0.023 | 0.00001 |
| YCR017C | CWH43 | 0.69439 | 0.018 | 1.02842 | 0.008 | -0.33539 | 0.018 | 0.00001 |
| YPL062W | YPL062W | 0.68888 | 0.01 | 1.03377 | 0.035 | -0.33447 | 0.01 | 0 |
| YNL190W | YNL190W | 0.64604 | 0.017 | 0.97827 | 0.017 | -0.33413 | 0.017 | 0 |
| YDR378C | LSM6 | 0.36328 | 0.019 | 0.68911 | 0.029 | -0.33339 | 0.019 | 0.00004 |
| YBL091C | MAP2 | 0.69635 | 0.012 | 1.02608 | 0.009 | -0.32975 | 0.012 | 0 |
| YCR073C | SSK22 | 0.75804 | 0.039 | 1.08594 | 0.02 | -0.32649 | 0.039 | 0.00004 |
| YBL070C | YBL070C | 0.71991 | 0.001 | 1.04991 | 0.026 | -0.32607 | 0.001 | 0 |
| YOR170W | YOR170W | 0.75075 | 0.033 | 1.08537 | 0.032 | -0.32589 | 0.033 | 0.00002 |
| YJL154C | VPS35 | 0.70954 | 0.009 | 1.02652 | 0.029 | -0.32458 | 0.009 | 0.00005 |
| YNL044W | YIP3 | 0.68573 | 0.027 | 1.00575 | 0.015 | -0.32299 | 0.027 | 0.00002 |
| YFR039C | YFR039C | 0.74921 | 0.028 | 1.0723 | 0.013 | -0.32171 | 0.028 | 0.00001 |
| YDL076C | RXT3 | 0.63462 | 0.033 | 0.95721 | 0.036 | -0.32164 | 0.033 | 0.00004 |
| YER167W | BCK2 | 0.71175 | 0.033 | 1.03183 | 0.011 | -0.31983 | 0.033 | 0.00003 |
| YLR342W | FKS1 | 0.65077 | 0.025 | 0.97067 | 0.002 | -0.31971 | 0.025 | 0.00002 |
| YGL262W | YGL262W | 0.71935 | 0.018 | 1.03931 | 0.003 | -0.31948 | 0.018 | 0 |
| YOR069W | VPS5 | 0.47773 | 0.035 | 0.79223 | 0.007 | -0.31831 | 0.035 | 0.00011 |
| YBL057C | PTH2 | 0.69312 | 0.023 | 1.01273 | 0.025 | -0.31796 | 0.023 | 0.00038 |
| YBR157C | ICS2 | 0.57028 | 0.024 | 0.89029 | 0.005 | -0.31785 | 0.024 | 0.00002 |
| YIR037W | HYR1 | 0.74906 | 0.031 | 1.05857 | 0.032 | -0.31765 | 0.031 | 0.00002 |
| YAL061W | BDH2 | 0.73276 | 0.035 | 1.05078 | 0.017 | -0.31577 | 0.035 | 0.00003 |
| YBL069W | AST1 | 0.74559 | 0.013 | 1.06336 | 0.019 | -0.31538 | 0.013 | 0 |
| YNL285W | YNL285W | 0.75604 | 0.013 | 1.0694 | 0.005 | -0.31493 | 0.013 | 0 |
| YPR020W | ATP20 | 0.69162 | 0.011 | 1.00382 | 0.004 | -0.31478 | 0.011 | 0 |
| YAL002W | VPS8 | 0.67674 | 0.024 | 0.98913 | 0.011 | -0.31458 | 0.024 | 0.00001 |
| YPR140W | TAZ1 | 0.76304 | 0.004 | 1.07544 | 0.01 | -0.31416 | 0.004 | 0.00001 |
| YOR068C | VAM10 | 0.67066 | 0.012 | 0.98359 | 0.006 | -0.31385 | 0.012 | 0 |
| YNL324W | YNL324W | 0.84377 | 0.018 | 1.15249 | 0.016 | -0.31361 | 0.018 | 0.00015 |
| YBL039C | URA7 | 0.73293 | 0.017 | 1.04753 | 0.003 | -0.31348 | 0.017 | 0.00017 |
| YBR204C | LDH1 | 0.70368 | 0.037 | 1.02141 | 0.017 | -0.31303 | 0.037 | 0.00093 |
| YLR408C | BLS1 | 0.7123 | 0.034 | 1.02892 | 0.024 | -0.31254 | 0.034 | 0.00003 |
| YDR290W | YDR290W | 0.58317 | 0.021 | 0.89682 | 0.009 | -0.31232 | 0.021 | 0.00001 |
| YNL168C | FMP41 | 0.81419 | 0.035 | 1.12535 | 0.022 | -0.30993 | 0.035 | 0.00002 |
| YBR071W | YBR071W | 0.67389 | 0.036 | 0.98606 | 0.029 | -0.30922 | 0.036 | 0.00004 |
| YFL049W | SWP82 | 0.6914 | 0.025 | 1.00001 | 0.012 | -0.30917 | 0.025 | 0.00001 |
| YPR093C | ASR1 | 0.67943 | 0.008 | 0.98682 | 0.042 | -0.30902 | 0.008 | 0 |
| YBR216C | YBP1 | 0.70175 | 0.019 | 1.01748 | 0.025 | -0.3083 | 0.019 | 0.00001 |
| YBR028C | YPK3 | 0.67141 | 0.014 | 0.97867 | 0.024 | -0.30783 | 0.014 | 0 |
| YEL042W | GDA1 | 0.71859 | 0.023 | 1.0316 | 0.027 | -0.30748 | 0.023 | 0.00001 |
| YGR100W | MDR1 | 0.66509 | 0.018 | 0.96564 | 0.03 | -0.30688 | 0.018 | 0.00001 |
| YGL059W | PKP2 | 0.86992 | 0.028 | 1.1792 | 0.005 | -0.30668 | 0.028 | 0.00033 |
| YKR104W | YKR104W | 0.77103 | 0.033 | 1.07402 | 0.007 | -0.30561 | 0.033 | 0.00002 |
| YNL035C | YNL035C | 0.85337 | 0.027 | 1.16109 | 0.113 | -0.30505 | 0.027 | 0.00001 |
| YIL154C | IMP2' | 0.66454 | 0.014 | 0.97322 | 0.007 | -0.30466 | 0.014 | 0 |
| YDL018C | ERP3 | 0.77277 | 0.02 | 1.07436 | 0.01 | -0.30408 | 0.02 | 0.00001 |
| YML042W | CAT2 | 0.77653 | 0.016 | 1.08357 | 0.01 | -0.30369 | 0.016 | 0.00014 |
| YEL017W | GTT3 | 0.71006 | 0.019 | 1.01232 | 0.001 | -0.30137 | 0.019 | 0.00001 |
| YGR254W | ENO1 | 1.44566 | 0.012 | 1.03525 | 0.009 | 0.40849 | 0.012 | 0.00002 |
| YIL101C | XBP1 | 1.35608 | 0.049 | 0.94866 | 0.022 | 0.41116 | 0.049 | 0.00001 |
| YJR119C | JHD2 | 1.47203 | 0.029 | 1.0629 | 0.02 | 0.4117 | 0.029 | 0 |
| YER182W | FMP10 | 1.41712 | 0.042 | 1.00539 | 0.005 | 0.4125 | 0.042 | 0.00001 |
| YEL071W | DLD3 | 1.4324 | 0.034 | 1.02002 | 0.025 | 0.41253 | 0.034 | 0 |
| YEL039C | CYC7 | 1.44995 | 0.186 | 1.03521 | 0.008 | 0.41336 | 0.186 | 0.00058 |
| YIL034C | CAP2 | 1.43512 | 0.2 | 1.01006 | 0.043 | 0.41346 | 0.2 | 0.00074 |
| YOL087C | DUF1 | 1.33173 | 0.003 | 0.92124 | 0.035 | 0.41481 | 0.003 | 0 |
| YKL198C | PTK1 | 1.42293 | 0.079 | 1.00549 | 0.023 | 0.41516 | 0.079 | 0.00005 |
| YGR184C | UBR1 | 1.38878 | 0.003 | 0.97302 | 0.006 | 0.41551 | 0.003 | 0 |
| YIL047C | SYG1 | 1.41705 | 0.058 | 1.00129 | 0.043 | 0.41713 | 0.058 | 0.00002 |
| YJL135W | YJL135W | 1.46684 | 0.038 | 1.04998 | 0.022 | 0.41889 | 0.038 | 0 |
| YAR042W | SWH1 | 1.39384 | 0.02 | 0.97962 | 0.012 | 0.41893 | 0.02 | 0.00007 |
| YDL155W | CLB3 | 1.51974 | 0.044 | 1.10111 | 0.008 | 0.41914 | 0.044 | 0.00001 |
| YKR100C | SKG1 | 1.41077 | 0.021 | 0.99177 | 0.007 | 0.42036 | 0.021 | 0.00007 |
| YOL007C | CSI2 | 1.38224 | 0.04 | 0.9608 | 0.017 | 0.42392 | 0.04 | 0.00001 |
| YML070W | DAK1 | 1.5033 | 0.015 | 1.07888 | 0.014 | 0.42394 | 0.015 | 0 |
| YDL053C | PBP4 | 1.43075 | 0.048 | 1.00657 | 0.002 | 0.42513 | 0.048 | 0.00001 |
| YMR274C | RCE1 | 1.33238 | 0.138 | 0.90877 | 0.006 | 0.42553 | 0.138 | 0.0003 |
| YDR371W | CTS2 | 1.29102 | 0.034 | 0.87134 | 0.016 | 0.42634 | 0.034 | 0.00001 |
| YPR027C | YPR027C | 1.51443 | 0.027 | 1.08507 | 0.011 | 0.42719 | 0.027 | 0 |
| YDR318W | MCM21 | 1.43785 | 0.045 | 1.00765 | 0.016 | 0.42782 | 0.045 | 0.00001 |
| YER163C | GCG1 | 1.41079 | 0.052 | 0.98386 | 0.016 | 0.42876 | 0.052 | 0.00001 |
| YFR023W | PES4 | 1.29806 | 0.036 | 0.86904 | 0.015 | 0.42877 | 0.036 | 0.00001 |
| YMR182C | RGM1 | 1.42071 | 0.022 | 0.98587 | 0.018 | 0.43497 | 0.022 | 0 |
| YGL104C | VPS73 | 1.49564 | 0.019 | 1.05909 | 0.012 | 0.4358 | 0.019 | 0.00005 |
| YNL157W | IGO1 | 1.46101 | 0.042 | 1.02059 | 0.011 | 0.4393 | 0.042 | 0.00001 |
| YBR245C | ISW1 | 1.42985 | 0.005 | 0.99259 | 0.014 | 0.44002 | 0.005 | 0 |
| YMR305C | SCW10 | 1.42818 | 0.199 | 0.98427 | 0.038 | 0.44149 | 0.199 | 0.00073 |
| YPR171W | BSP1 | 1.54873 | 0.066 | 1.09882 | 0.024 | 0.44651 | 0.066 | 0.00002 |
| YMR153C-A | YMR153C-A | 1.46262 | 0.031 | 1.01495 | 0.018 | 0.44792 | 0.031 | 0 |
| YNL024C | YNL024C | 1.39756 | 0.11 | 0.94744 | 0.006 | 0.45063 | 0.11 | 0.00013 |
| YIL117C | PRM5 | 1.42992 | 0.073 | 0.98102 | 0.005 | 0.45175 | 0.073 | 0.00004 |
| YDR095C | YDR095C | 1.44815 | 0.009 | 0.99775 | 0.003 | 0.45208 | 0.009 | 0.00001 |
| YCR106W | RDS1 | 1.45809 | 0.01 | 1.01121 | 0.024 | 0.45471 | 0.01 | 0 |
| YOR289W | YOR289W | 1.48179 | 0.058 | 1.02476 | 0.011 | 0.45658 | 0.058 | 0.00002 |
| YMR135C | GID8 | 1.45356 | 0.01 | 0.9972 | 0.002 | 0.4566 | 0.01 | 0.00002 |
| YDL154W | MSH5 | 1.48116 | 0.233 | 1.0276 | 0.036 | 0.45787 | 0.233 | 0.00105 |
| YPL064C | CWC27 | 1.46312 | 0.025 | 1.0097 | 0.02 | 0.45797 | 0.025 | 0.0001 |
| YGL067W | NPY1 | 1.49678 | 0.021 | 1.03151 | 0.021 | 0.46662 | 0.021 | 0 |
| YGL249W | ZIP2 | 1.50077 | 0.089 | 1.03709 | 0.015 | 0.46677 | 0.089 | 0.00006 |
| YPL229W | YPL229W | 1.53327 | 0.004 | 1.05905 | 0.016 | 0.47358 | 0.004 | 0 |
| YGL232W | TAN1 | 1.4709 | 0.046 | 0.99046 | 0.026 | 0.47716 | 0.046 | 0.00001 |
| YNR045W | PET494 | 1.43977 | 0.202 | 0.95552 | 0.04 | 0.47981 | 0.202 | 0.00074 |
| YDL224C | WHI4 | 1.5391 | 0.009 | 1.056 | 0.004 | 0.48333 | 0.009 | 0.00001 |
| YGR069W | YGR069W | 1.50637 | 0.197 | 1.02006 | 0.008 | 0.4873 | 0.197 | 0.00061 |
| YGL263W | COS12 | 1.49684 | 0.017 | 1.01012 | 0.006 | 0.48792 | 0.017 | 0 |
| YIR019C | FLO11 | 1.50721 | 0.102 | 1.02648 | 0.037 | 0.4882 | 0.102 | 0.00008 |
| YLR391W | YLR391W | 1.53535 | 0.038 | 1.05212 | 0.031 | 0.48955 | 0.038 | 0 |
| YER080W | AIM9 | 1.54853 | 0.05 | 1.0605 | 0.015 | 0.4914 | 0.05 | 0.00001 |
| YGR192C | TDH3 | 1.46289 | 0.01 | 0.97356 | 0.018 | 0.49395 | 0.01 | 0 |
| YBR181C | RPS6B | 1.42366 | 0.072 | 0.93223 | 0.011 | 0.49478 | 0.072 | 0.00004 |
| YAR043C | YAR043C | 1.44978 | 0.027 | 0.95447 | 0.013 | 0.49507 | 0.027 | 0 |
| YIL138C | TPM2 | 1.54775 | 0.018 | 1.05685 | 0.028 | 0.49587 | 0.018 | 0 |
| YOR051C | ETT1 | 1.47674 | 0.034 | 0.97967 | 0.002 | 0.4971 | 0.034 | 0 |
| YDL170W | UGA3 | 1.53664 | 0.025 | 1.03731 | 0.007 | 0.4991 | 0.025 | 0 |
| YGR071C | ENV11 | 1.52277 | 0.071 | 1.02128 | 0.01 | 0.50035 | 0.071 | 0.00003 |
| YOR183W | FYV12 | 1.37857 | 0.031 | 0.87481 | 0.008 | 0.50242 | 0.031 | 0.00017 |
| YAL030W | SNC1 | 1.64154 | 0.055 | 1.13478 | 0.012 | 0.50786 | 0.055 | 0.00001 |
| YKL066W | YKL066W | 1.51049 | 0.018 | 1.001 | 0.015 | 0.50857 | 0.018 | 0 |
| YOR144C | ELG1 | 1.56347 | 0.022 | 1.05057 | 0.006 | 0.50993 | 0.022 | 0 |
| YFR018C | YFR018C | 1.51745 | 0.04 | 1.01196 | 0.02 | 0.51113 | 0.04 | 0.00023 |
| YAL011W | SWC3 | 1.44411 | 0.026 | 0.93089 | 0.008 | 0.51508 | 0.026 | 0 |
| YBR040W | FIG1 | 1.56895 | 0.032 | 1.0603 | 0.014 | 0.51633 | 0.032 | 0 |
| YJR005W | APL1 | 1.53403 | 0.047 | 1.02152 | 0.014 | 0.51687 | 0.047 | 0.00001 |
| YIL170W | HXT12 | 1.53844 | 0.06 | 1.01334 | 0.017 | 0.52138 | 0.06 | 0.00002 |
| YMR304C-A | YMR304C-A | 1.52159 | 0.012 | 0.99247 | 0.014 | 0.52743 | 0.012 | 0.00002 |
| YBL075C | SSA3 | 1.56987 | 0.006 | 1.03865 | 0.009 | 0.53637 | 0.006 | 0 |
| YBR205W | KTR3 | 1.6109 | 0.047 | 1.07257 | 0.016 | 0.54173 | 0.047 | 0.00001 |
| YBR162W-A | YSY6 | 1.56506 | 0.009 | 1.02038 | 0.014 | 0.5475 | 0.009 | 0 |
| YNR060W | FRE4 | 1.67467 | 0.045 | 1.13269 | 0.032 | 0.54969 | 0.045 | 0.00001 |
| YNL283C | WSC2 | 1.58087 | 0.019 | 1.02942 | 0.013 | 0.55049 | 0.019 | 0 |
| YNR057C | BIO4 | 1.63259 | 0.025 | 1.08009 | 0.01 | 0.55433 | 0.025 | 0 |
| YER096W | SHC1 | 1.60375 | 0.048 | 1.0344 | 0.008 | 0.57177 | 0.048 | 0.00001 |
| YDR380W | ARO10 | 1.62868 | 0.01 | 1.05471 | 0.006 | 0.57584 | 0.01 | 0.00001 |
| YIL122W | POG1 | 1.67166 | 0.001 | 1.09141 | 0.014 | 0.57849 | 0.001 | 0 |
| YAL004W | YAL004W | 1.60076 | 0.049 | 1.0152 | 0.009 | 0.58613 | 0.049 | 0.00001 |
| YIL100W | YIL100W | 1.58441 | 0.01 | 0.99608 | 0.018 | 0.59124 | 0.01 | 0 |
| YDR133C | YDR133C | 1.71373 | 0.081 | 1.09482 | 0.035 | 0.62052 | 0.081 | 0.00003 |
| YBL024W | NCL1 | 1.6025 | 0.041 | 0.97099 | 0.003 | 0.63444 | 0.041 | 0 |
| YGL090W | LIF1 | 1.67456 | 0.037 | 1.02109 | 0.001 | 0.65423 | 0.037 | 0 |
| YBL019W | APN2 | 1.64727 | 0.079 | 0.99282 | 0.006 | 0.65975 | 0.079 | 0.00003 |
| YGL176C | YGL176C | 1.75284 | 0.1 | 1.0789 | 0.053 | 0.66176 | 0.1 | 0.00005 |
| YDL121C | YDL121C | 1.70892 | 0.085 | 1.04494 | 0.011 | 0.66337 | 0.085 | 0.00003 |
| YGL163C | RAD54 | 1.68943 | 0.065 | 0.99994 | 0.012 | 0.68774 | 0.065 | 0.00002 |
| YBR131W | CCZ1 | 1.77283 | 0.038 | 1.02583 | 0.011 | 0.74863 | 0.038 | 0 |
| YDR096W | GIS1 | 1.86044 | 0.002 | 1.07109 | 0.03 | 0.79903 | 0.002 | 0 |
